# CD28 costimulation drives tumor-infiltrating T cell glycolysis to promote inflammation

**DOI:** 10.1172/jci.insight.138729

**Published:** 2020-08-20

**Authors:** Kathryn E. Beckermann, Rachel Hongo, Xiang Ye, Kirsten Young, Katie Carbonell, Diana C. Contreras Healey, Peter J. Siska, Sierra Barone, Caroline E. Roe, Christof C. Smith, Benjamin G. Vincent, Frank M. Mason, Jonathan M. Irish, W. Kimryn Rathmell, Jeffrey C. Rathmell

**Affiliations:** 1 Department of Medicine, Division of Hematology and Oncology, and; 2 Department of Pathology, Microbiology, and Immunology, Vanderbilt University Medical Center, Nashville, Tennessee, USA.; 3 Internal Medicine III, University Hospital Regensburg, Regensburg, Germany.; 4 Department of Cell and Developmental Biology, Vanderbilt University School of Medicine, Nashville, Tennessee, USA.; 5 Lineberger Comprehensive Cancer Center; Department of Medicine Division of Hematology and Oncology, Department of Microbiology and Immunology, Curriculum in Bioinformatics and Computational Biology, Computational Medicine Program, University of North Carolina (UNC), Chapel Hill, North Carolina, USA.; 6 Center for Immunobiology, Vanderbilt University Medical Center, Nashville, Tennessee, USA.

**Keywords:** Immunology, Oncology, Glucose metabolism, Immunotherapy, T cells

## Abstract

Metabolic reprogramming dictates the fate and function of stimulated T cells, yet these pathways can be suppressed in T cells in tumor microenvironments. We previously showed that glycolytic and mitochondrial adaptations directly contribute to reducing the effector function of renal cell carcinoma (RCC) CD8^+^ tumor-infiltrating lymphocytes (TILs). Here we define the role of these metabolic pathways in the activation and effector functions of CD8^+^ RCC TILs. CD28 costimulation plays a key role in augmenting T cell activation and metabolism, and is antagonized by the inhibitory and checkpoint immunotherapy receptors CTLA4 and PD-1. While RCC CD8^+^ TILs were activated at a low level when stimulated through the T cell receptor alone, addition of CD28 costimulation greatly enhanced activation, function, and proliferation. CD28 costimulation reprogrammed RCC CD8^+^ TIL metabolism with increased glycolysis and mitochondrial oxidative metabolism, possibly through upregulation of GLUT3. Mitochondria also fused to a greater degree, with higher membrane potential and overall mass. These phenotypes were dependent on glucose metabolism, as the glycolytic inhibitor 2-deoxyglucose both prevented changes to mitochondria and suppressed RCC CD8^+^ TIL activation and function. These data show that CD28 costimulation can restore RCC CD8^+^ TIL metabolism and function through rescue of T cell glycolysis that supports mitochondrial mass and activity.

## Abstract 2

T cells in renal cell carcinomas develop metabolic barriers to inflammatory function that can be overcome by costimulation.

## Introduction

Glucose is a fundamental anabolic nutrient for proliferating cells (1). It is also now clear that stimulated T cells are highly dependent on metabolic reprogramming from a catabolic oxidative metabolism to an anabolic metabolism with elevated glucose consumption and aerobic glycolysis to develop effector function (2–4). T cell activation leads to increased Myc and PI3K/Akt/mTORC1 signaling activity to promote glucose uptake and mitochondrial metabolism for growth, energetics, and to regulate signaling and gene expression pathways (5–8). These changes are critical for effector T cell function, as T cells deficient in the glucose transporter GLUT1 or subject to inhibition of glucose metabolism fail to develop into effector subsets, have reduced capacity to induce inflammatory diseases, and instead favor suppressive Treg fates (9). Conversely, increased glucose metabolism can enhance effector T cell function and inflammation (10), and compensatory increases in glycolysis can allow inhibition of glutamine metabolism to also increase effector function (11, 12).

Costimulatory and coinhibitory pathways are critical regulators of T cell metabolism. Activation through the T cell receptor alone fails to induce metabolic reprogramming and can instead lead to a metabolically quiescent anergic state (5, 13). Activation together with signaling through the costimulatory molecule CD28, however, augments signaling through the PI3K/Akt/mTORC1 pathway to increase glucose and mitochondrial metabolism and enable robust proliferation and effector function (7). CD28 costimulation can increase T cell anabolic metabolism, while the CD28 family members PD-1 and CTLA4 suppress T cell metabolic reprogramming. PD-1 inhibits glycolysis and instead can promote fatty acid oxidation (FAO) of endogenous lipids and alter nucleoside synthesis. PD-1 can also negatively regulate T cells through changes in formation of mitochondrial cristae, which serve to impair oxidative phosphorylation (14). In addition, CTLA4 acts to inhibit CD28 signaling and PI3K/Akt/mTORC1 signaling, resulting in decreased glycolysis and mitochondrial oxidative capacity (15, 16).

The T cell requirement for anabolic metabolic reprogramming can render effector T cells susceptible to microenvironmental restrictions or metabolic pressures that negatively impact T cell activation and function. The metabolism and microenvironment of tumors coupled with chronic exposure to neoantigens in particular can impair the metabolism of tumor-infiltrating lymphocytes (TILs) (17–19). Indeed, T cells in tumors can be subject to metabolic barriers that lead to adaptations such as reduced glucose and mitochondrial metabolism with mitochondrial fragmentation and impairment (18–23). PGC1α is important for mitochondrial biogenesis, and its progressive loss in TIL may contribute to metabolic fitness of TILs (19). Overcoming these metabolic barriers by direct provision of nutrients, including the mitochondrial fuels pyruvate or acetate, or by genetically restoring mitochondrial function, can improve TIL effector function (18–23). These studies demonstrate a direct inhibitory role of impaired TIL metabolism on TIL effector functionality.

Renal cell carcinoma (RCC) is well known to be sensitive to T cell–directed immune therapy (24). RCC is associated with mutations in the von Hippel Lindau (*VHL*) gene, which lead to stabilization of hypoxia-inducible factor–1α (HIF1A) and higher rates of aerobic glycolysis than in normal tissue (25, 26). While RCC can respond to PD-1 and CTLA4 checkpoint blockade immunotherapy, only a portion of patients achieve partial responses, and a smaller fraction generates durable responses (27–29). The immunological atlas of RCC has been defined, and T cells are abundant; unlike in most cancers, however, increased T cell infiltration is not associated with improved survival in RCC. We previously showed that CD8^+^ TILs from patients with RCC had widespread metabolic deficiencies (18, 30). These defects included decreased ability to take up glucose for downstream glycolysis, and fragmented and functionally altered mitochondria with low respiratory capacity and elevated production of ROS (18). The role of these metabolic adaptations and costimulatory pathways in RCC CD8^+^ TIL activation, however, remains uncertain.

Here we assess the role of costimulation in T cell metabolism and effector function in the RCC tumor microenvironment. RCC CD8^+^ TILs had altered metabolic and functional parameters, suggesting reduced metabolism and the failure of antigen receptor stimulation to activate a predominant effector memory phenotype. Consistent with responses in RCC to checkpoint blockade therapy and the role of CTLA4 in inhibiting CD28 signaling, a large portion of RCC CD8^+^ TILs had greatly elevated markers of activation and functional capacity when activated through the T cell receptor together with CD28 costimulation. Mechanistically, CD28-costimulated RCC CD8^+^ TILs also showed increased anabolic glycolysis and mitochondrial metabolism, both of which were critically maintained by glucose metabolism, as the glycolytic inhibitor 2-deoxyglucose suppressed TIL mitochondrial fusion and accumulation, as well as activation and proliferation. These data show that a large portion of RCC CD8^+^ TILs can restore metabolic and functional parameters when provided adequate costimulation, and that glucose metabolism is required for rescue of mitochondrial status and inflammatory functions.

## Results

### CD8^+^ RCC TILs exhibit differential inhibitory signaling and metabolic pathways.

**Figure 1:**
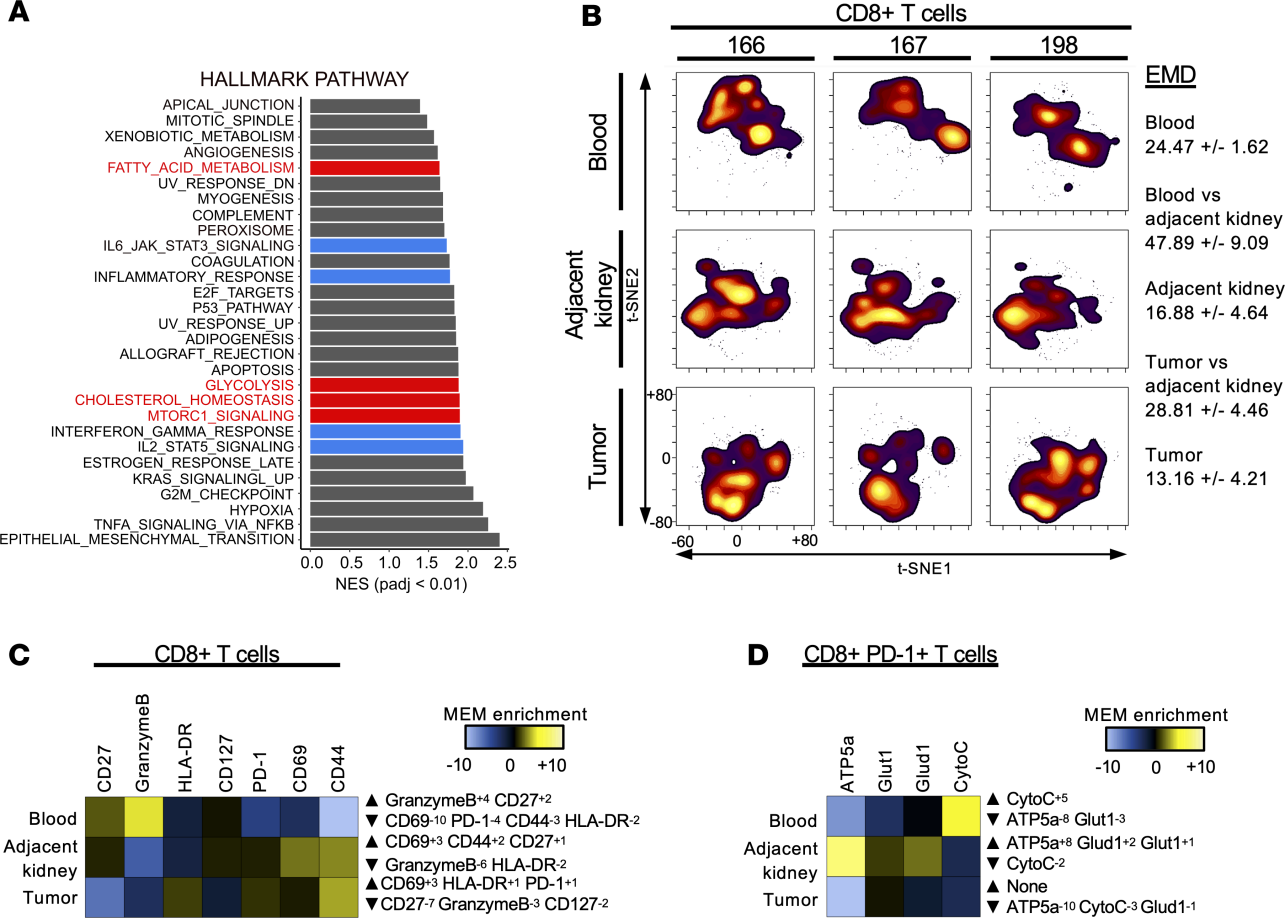
RCC CD8^+^ TILs differentially express costimulatory, checkpoint inhibitor, and metabolic pathways compared with matched peripheral blood CD8^+^ cells. (**A**) Gene set enrichment analysis was performed, and enrichment scores are shown for pathway enrichment in CD8^+^ RCC TILs compared with peripheral blood. Red highlights enriched metabolic pathways. Blue highlights T cell effector signaling pathways. Normalized enrichment scores (NES) are shown for pathways with BH-adjusted *P* < 0.01. *n* = 5. (**B**) t-SNE analysis of 3 independent patients with RCC showing matching patient peripheral blood, RCC tumor, and adjacent kidney tissue. Average EMD (*n* = 3) compared across sample types: blood versus adjacent kidney, and tumor versus adjacent kidney. (**C**) MEM used to quantitatively determine the phenotype of CD8^+^ T cells for patients 166, 167, and 198 within a given tissue type as compared with all other samples. (**D**) MEM applied to assess CD8^+^PD-1^+^ cells, determining metabolic phenotype across all samples. CytoC, cytochrome *c*.

To investigate pathways regulating T cell function and metabolism in the RCC tumor microenvironment, CD8^+^ T cells were sorted from the tumors of 5 patients with RCC, and gene expression was compared with that in matched patient peripheral blood CD8^+^ T cells. Analysis of paired RNA-Seq data by gene set enrichment of differentially expressed genes showed that RCC CD8^+^ TILs had striking differences from control CD8^+^ T cells. RCC CD8^+^ TILs were enriched for expression of gene signatures of both inflammatory effector function and altered metabolism (Figure 1A). In addition to altered cytokine and inflammatory signaling pathways, metabolic changes included altered glycolysis and lipid metabolism, which suggested changes to both glucose and mitochondrial metabolism. Gene set enrichment analyses showed that RCC CD8^+^ TILs had increased expression of multiple metabolic pathways (Supplemental Figure 1B; supplemental material available online with this article; https://doi.org/10.1172/jci.insight.138729DS1). CIBERSORT was used to group TIL subsets based on previously defined CD8^+^ T cell gene expression profiles (31). Tumor-associated CD8^+^ T cells had few naive-subset CD8^+^ T cells and a higher percentage of cells categorized as exhausted compared with the matched peripheral blood of patients (Supplemental Figure 1A).

The immune and metabolic phenotypes of RCC CD8^+^ TILs were next compared by evaluating protein levels in matched tumors, normal tumor adjacent kidney, and peripheral blood by mass cytometry. A custom metal-labeled antibody panel was developed to simultaneously phenotype these samples, detect immune activation, and measure activity at the single-cell level. Metabolic markers included GLUT1, a glucose transporter; ATP5a, subunit of ATP synthase in oxidative phosphorylation; cytochrome *c*, which functions to transfer electrons in the mitochondrial respiratory chain; and GLUD1, involved in amino acid metabolism function and glutaminolysis. Data were analyzed using t-distributed stochastic neighbor embedding (t-SNE) dimensionality reduction and showed clear differences across 3 patients for the phenotypes of CD8^+^ T cells between each tissue (Figure 1B). Earth mover’s distance (EMD) was applied to quantitate the degree of difference of CD8^+^ T cells within each tissue and across tissues of the 3 patients (32). While the largest differences existed between T cells from patient blood and kidney tissue, the differences in RCC CD8^+^ TILs compared with matched adjacent kidney tissue were greater than those across the 3 tumors, demonstrating a phenotype distinct from that of kidney tissue–resident T cells. Marker enrichment modeling (MEM) showed that immunological markers of RCC CD8^+^ TILs were characterized by elevated PD-1 and reduced CD127 (Figure 1C) (33). A similar analysis of metabolic markers showed that RCC CD8^+^ TILs had reduced expression of markers of mitochondrial electron transport and metabolism (Figure 1D).

### Costimulation by CD28 rescues RCC CD8^+^ TIL effector function.

Because costimulatory and inhibitory receptors play critical roles in promoting or suppressing T cell metabolism, we examined expression of costimulatory and coinhibitory receptors in RCC CD8^+^ TILs. CD28 was abundant in both peripheral and TIL CD8^+^ T cells, and based on the role of CD28 costimulation in promoting T cell glucose uptake and metabolism, we focused our analysis on this costimulation method. Further, CTLA4 blockade has been demonstrated to inhibit CD28 signaling (5, 7, 34). Overall, RCC CD8^+^ TILs showed increased expression of many costimulatory and coinhibitory genes (PD-1, CTLA4, LAG3, TIM-3) and markers of activation 4-1BB [TNFRSF9], OX40L [TNFSF4], and CD70 [ligand of CD27]), as well as downregulation of CD3, CD8, and OX40 receptor (TNFRSF4) (Supplemental Figure 1C). We assessed CD28 expression at the protein level by flow cytometry and found that CD28 expression was present on a subset of CD8^+^ TILs and not significantly different from that in patient peripheral blood (Supplemental Figure 1D).

**Figure 2:**
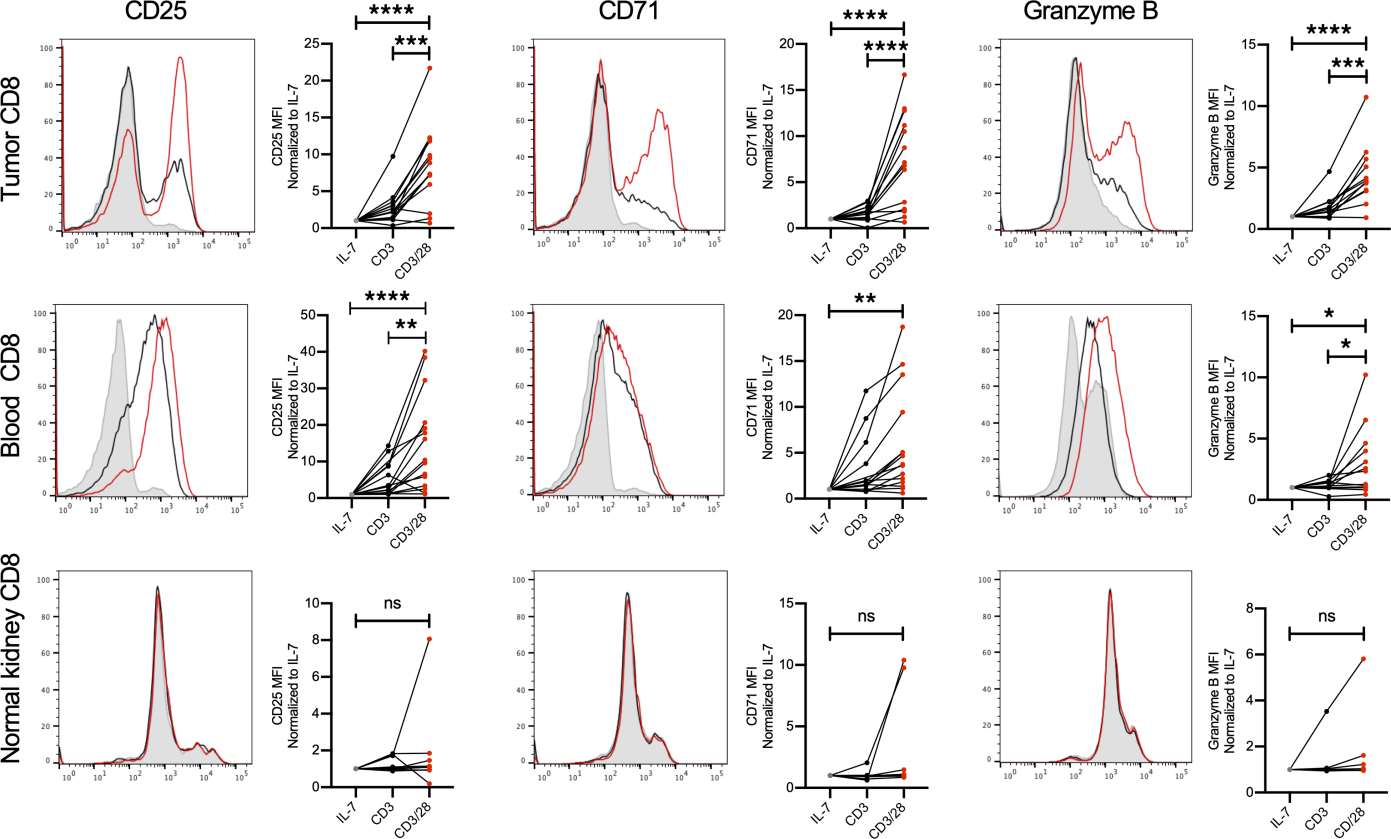
CD28 costimulation increases RCC CD8^+^ TIL activation and markers for effector function. Single-cell suspensions were cultured with IL-7 (gray) to maintain homeostasis, CD3 alone (black) for TCR engagement, or CD3 and CD28 costimulation (red) for 5 days before being subjected to flow cytometry. We assessed CD8^+^ RCC TILs, CD8^+^ cells from matched peripheral blood, and adjacent kidney CD8^+^ cells for markers of activation (CD25 and CD71) as well as effector function (granzyme B). *n* ≥ 13 patient blood and TIL; *n* ≥ 7 adjacent kidney tissue) *P < 0.05, **P < 0.01, ***P < 0.001, ****P < 0.0001 by 1-way ANOVA with Tukey’s post hoc test.

We tested whether costimulation with CD28 could overcome the impaired activation profile previously seen in RCC TILs. CD8^+^ cells from patient peripheral blood, matched adjacent kidney tissue, and RCC TILs were cultured in IL-7 to maintain viability in an unstimulated state, stimulated with anti-CD3 to stimulate the T cell receptor alone, or treated with anti-CD3 in combination with anti-CD28. As previously shown, T cell receptor engagement alone failed to broadly increase the surface markers of activation CD25 and CD71 and effector function as measured by granzyme B (Figure 2). However, addition of CD28 costimulation significantly enhanced the activation of peripheral blood and RCC TIL CD8^+^ T cells. Notably, while RCC TIL activation with anti-CD3 and CD28 costimulation did not occur upon stimulation in every patient, this response was evident in the majority of patient RCC TIL samples that were tested. In comparison, CD8^+^ T cells from adjacent matched kidney tissue had a previously activated phenotype and did not demonstrate enhancement in markers of activation or effector function when treated in these conditions (35). CD8^+^ T cells from peripheral blood of healthy donors followed the same pattern following stimulation (Supplemental Figure 2). These data demonstrate that a large portion of CD8^+^ T cells in many RCC patients remain capable of inducing effector functions when provided adequate CD28 costimulation.

### TIL populations define metabolic states when stimulated.

**Figure 3:**
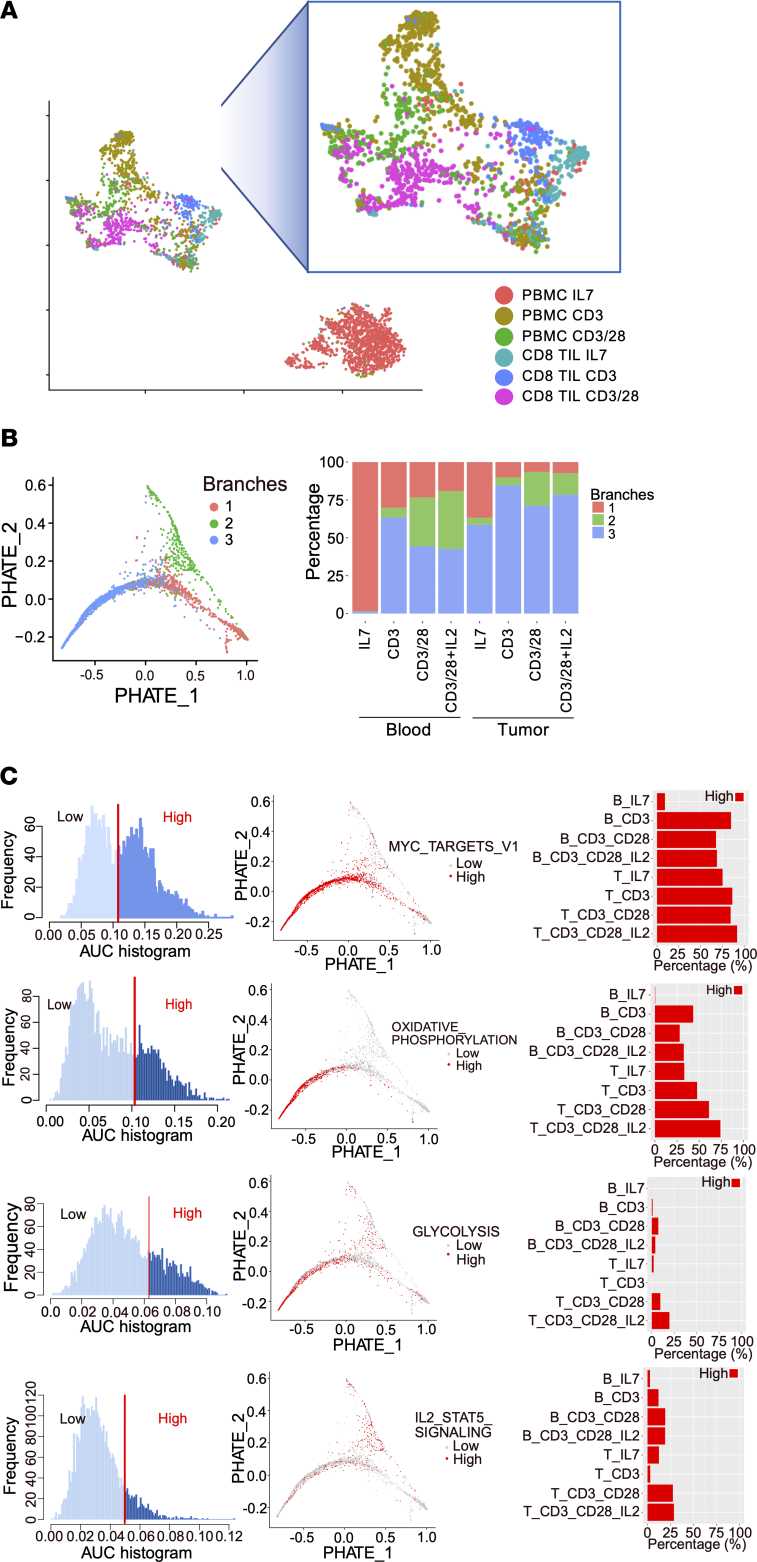
Single-cell gene expression analysis shows that CD28 costimulation increases CD8^+^ RCC TIL activity and metabolism. (**A**) UMAP analysis of single-cell RNA-Seq analysis of CD8 from peripheral blood and RCC TILs showing each sample treated with IL-7, CD3 alone, and CD3 with CD28 costimulation. (**B**) PHATE and monocle analysis using gene expression matrix revealed 2 distinct trajectories (green and blue) stemming from resting CD8^+^ T cells (red). Branches 1 (red), 2 (green), and 3 (blue) represent the 2 trajectories and the root resting state. Percentages of cells assigned to each branch in each sample are shown on the right. (**C**) Top pathways from hallmark gene sets that distinguish the 2 trajectories by pathway activities (AUC score). Pathway activities (AUCell score) for all cells are shown in the left panel as histogram by AUC score; pathway activity in cells past the threshold (vertical red line) was placed on the PHATE map trajectory (middle panel), with high-activity cells in red and low-activity cells in gray; bar graphs show the percentages of cells in each treatment that have high activity in each pathway.

Because TIL populations are heterogeneous, single-cell gene expression was employed to better identify pathways associated with increased RCC CD8^+^ TIL effector function following T cell receptor engagement and CD28 costimulation. T cells from control healthy donor PBMCs and RCC single-cell suspensions were examined following 5 days in culture to compare single-cell gene expression from T cells treated with IL-7 to maintain viability or stimulated with CD3 alone; CD3 with CD28; or CD3 with CD28 and IL-2 (Figure 3). The distance between cells reflects the difference in corresponding gene expression patterns analyzed by uniform manifold approximation and projection (UMAP) dimensionality reduction. The greatest difference in gene expression profiles was seen between unstimulated healthy donor control blood CD8^+^ cells treated only with maintenance IL-7 as compared with all other groups, including stimulated CD8^+^ PBMCs and all CD8^+^ TIL conditions (Figure 3A). CD8^+^ TILs that were cultured only with IL-7 showed some similarity to CD8^+^ T cells from control blood treated with CD3 and showed only modest differences from CD8^+^ TILs treated with CD3 alone. The addition of CD28 costimulation to CD3 stimulation, however, caused PBMC control and TIL CD8^+^ T cell populations to develop similar phenotypes.

We next analyzed single-cell RNA sequence data using PHATE, a visualization method that captures local and global nonlinear structure using information-geometric distance between data points (Figure 3B) (36). This analysis showed that T cells from each condition could be assigned to 3 distinct population branches, which is consistent with monocle analysis revealing 3 distinct states. Each of these population trajectories was characterized by a distinct gene expression signature (Figure 3C). Gene set enrichment analysis established branches, labeled resting (branch 1), IL-2 signaling and glycolysis (branch 2), and oxidative phosphorylation (branch 3). The IL-7–treated PBMC T cells existed entirely in the resting branch, while stimulated PBMCs entered either the IL-2 signaling and glycolysis or oxidative phosphorylation branches, with the IL-2 and glycolysis branch particularly enriched in cells treated with costimulation and IL-2 cytokine. In RCC TILs treated with control IL-7, the majority of cells were in the oxidative phosphorylation branch and the remainder in the resting branch, most similar to PBMC T cells treated with anti-CD3. In RCC TILs, CD28 costimulation led to increased representation in the IL-2 signaling branch, but to a lesser extent than in similarly treated PBMC T cells. The oxidative phosphorylation branch had enrichment in metabolic-associated pathways including oxidative phosphorylation, glycolysis, and Myc signatures, while the IL-2 signaling branch associated with pathways including glycolysis and IL-2/Stat5 signaling. Consistent with these gene signatures, the inhibitory receptors LAG3 and CTLA4 were preferentially expressed in the oxidative phosphorylation and IL-2 signaling branches compared with the resting branch (Supplemental Figure 3). Interestingly, the glucose transporter GLUT1 (SLC2A1) was expressed at low levels, while GLUT3 (SLC2A3) was induced primarily in the IL-2 signaling branch. In contrast, the oxidative phosphorylation signature was strongly supported by COX7C and ATP5E. Together, these data define genetic signatures of resting and 2 functionally and metabolically distinct activation states for CD8^+^ TILs, including a distinction between GLUT3 and oxidative phosphorylation populations.

**Figure 4:**
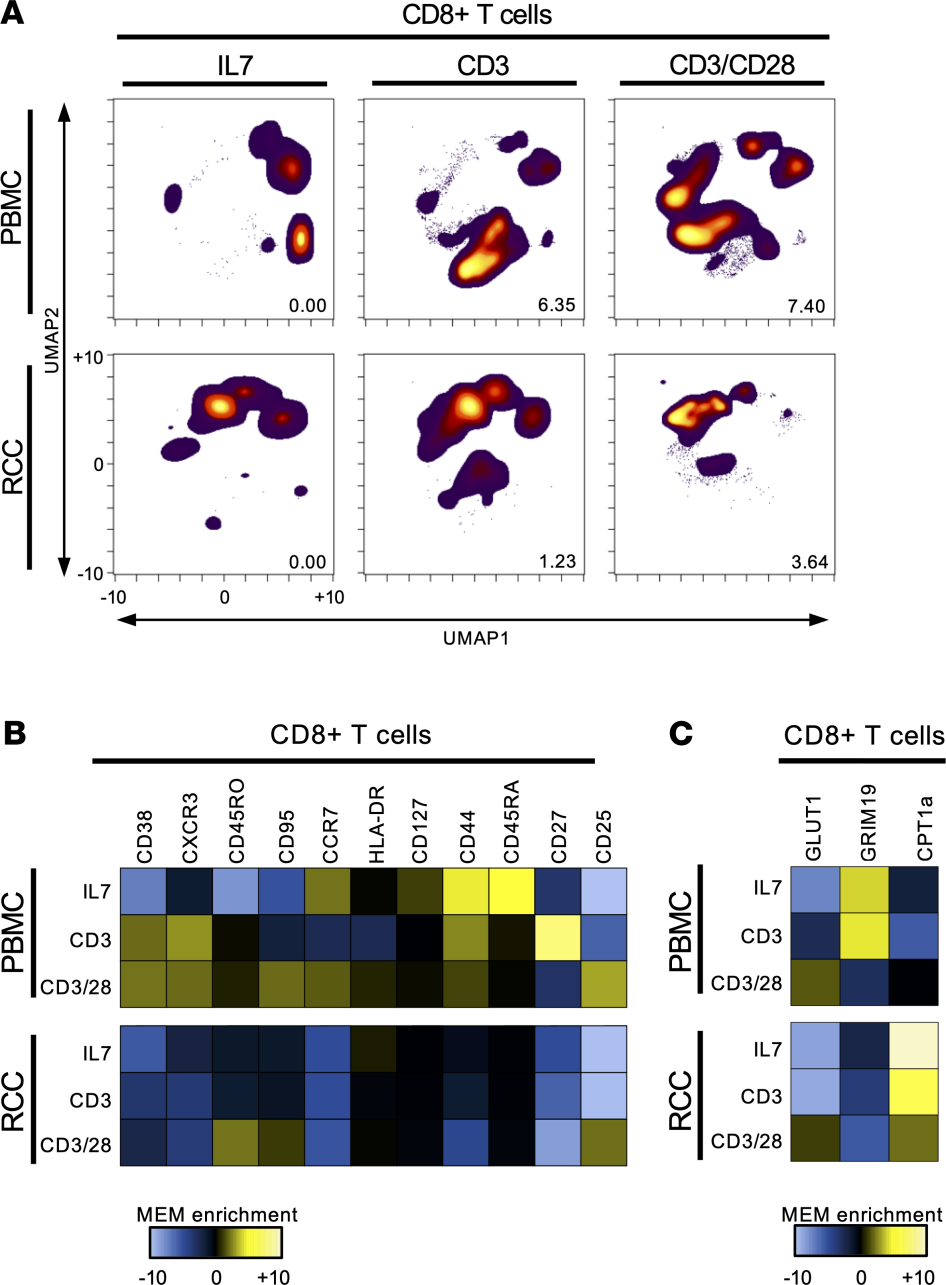
CD28 costimulation increases CD8^+^ TIL activity and metabolism. (**A**) UMAP of CD8^+^ T cells from healthy donor PBMCs and RCC TILs using mass cytometry analysis after 5 days of treatment with IL-7, CD3 alone, or CD3 with CD28 costimulation. (**B**) MEM used to quantitatively determine the phenotype of CD8^+^ T cells from healthy donor PBMCs and RCC CD8^+^ TILs following treatment with IL-7, CD3 alone, or CD3 with CD28 costimulation. (**C**) MEM applied to assess the metabolic phenotype of CD8^+^ T cells from healthy donor PBMCs and RCC TILs treated with IL-7, CD3 alone, or CD3 with CD28 costimulation.

The gene expression changes of CD28-costimulated CD8^+^ RCC TILs suggested enhanced activation with IL-2/Stat5 signaling and glycolysis. To determine how these gene expression changes affected TIL protein expression, activation, and metabolism, we performed CyTOF on healthy donor PBMC or RCC TIL treated with IL-7, CD3, and CD3 with CD28. As expected, CD8^+^ T cells in the healthy donor peripheral blood showed the biggest difference by UMAP analysis and EMD calculated between the IL-7 treatment and stimulation with CD3 alone, while in the RCC CD8^+^ TIL a bigger shifted was noted after treatment with combined CD3 and CD28 costimulation (Figure 4A). While anti-CD3 alone had minimal effect on CD8^+^ RCC TIL, addition of CD28 costimulation led to a significant shift in T cell phenotype with enrichment of CD25, CD95, and CD45RO (Figure 4B). Metabolic markers were also altered in RCC CD8^+^ TILs, as demonstrated by CyTOF (Figure 4C). The mitochondrial electron transport protein GRIM19 was expressed at low levels, while the mitochondrial enzyme CPT1A was expressed at high levels in RCC CD8^+^ TILs relative to peripheral blood CD8^+^ T cells. Following activation and CD28 costimulation, both GRIM19 and CPT1A decreased, while GLUT1 expression was similar or slightly increased.

### CD28 costimulation of RCC CD8^+^ TILs increases glycolytic and mitochondrial metabolism.

**Figure 5:**
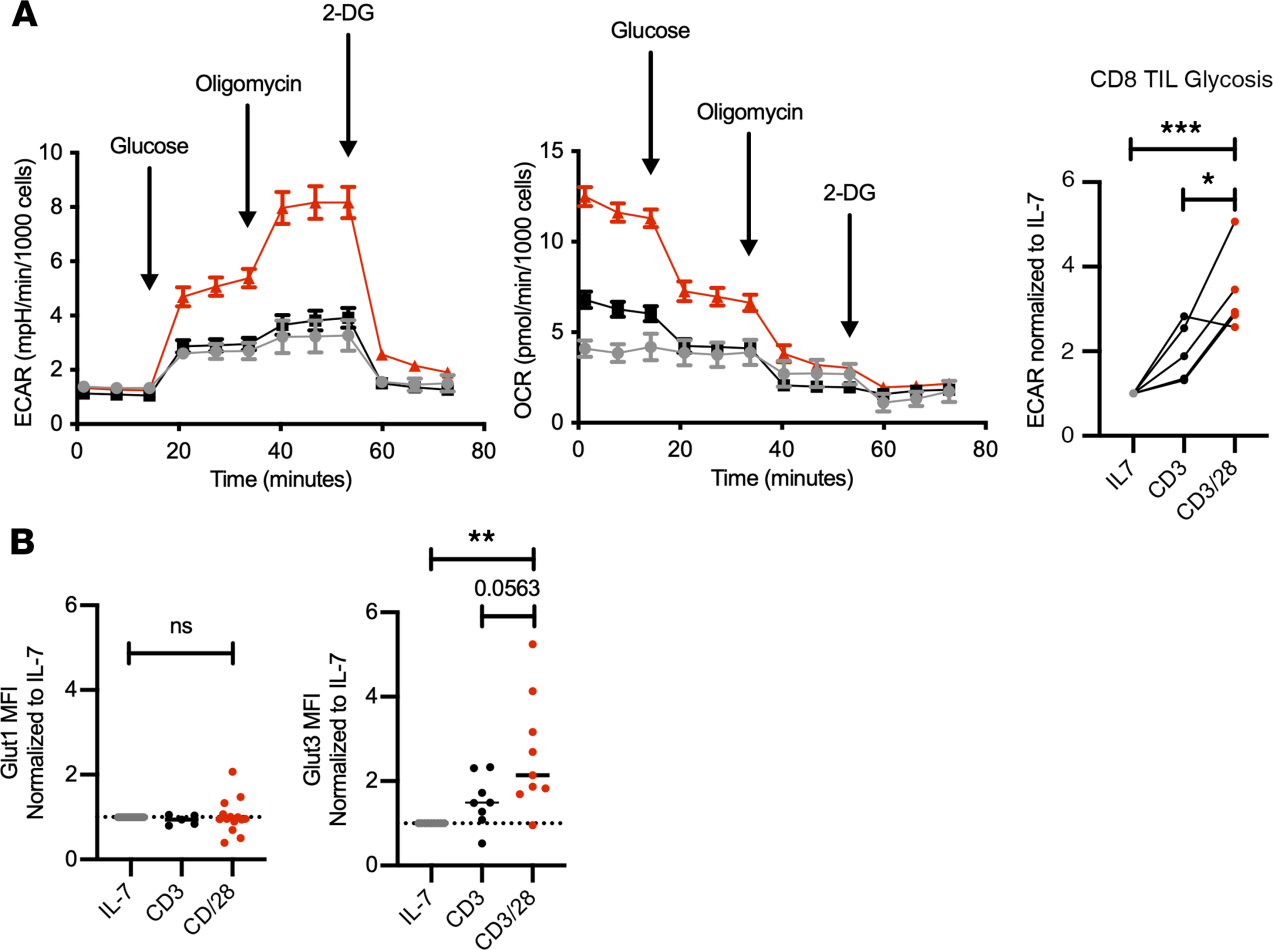
CD28 costimulation increases glycolysis and glucose transporters. (**A**) Glycolytic stress test results showing representative ECAR and OCR (±SEM) normalized to cell count using Cytation 5 (BioTek). CD8^+^ RCC TIL glycolysis following IL-7 (gray), CD3 alone (black), CD3 with CD28 costimulation (red). *n* = 5. *P < 0.05, ***P < 0.001 by 1-way ANOVA with Tukey’s post hoc test. (**B**) Flow cytometry analysis showing MFI of GLUT1 and GLUT3 normalized to IL-7 and comparing CD3 with CD28 costimulation (red). *n* ≥ 9. **P < 0.01 by 1-way ANOVA with Tukey’s post hoc test.

Gene expression and protein analyses suggested that RCC CD8^+^ TILs had reduced glycolysis and mitochondrial metabolism that could be restored with CD28 costimulation. For direct testing of the role of costimulation in RCC CD8^+^ TIL metabolism, T cells were isolated and cultured in IL-7 to remain in a resting state or in anti-CD3 either alone or in combination with anti-CD28. Extracellular flux analyses to measure metabolic parameters found that CD28 costimulation led to a sharp increase in both glycolysis as measured by extracellular acidification rate (ECAR) and oxygen consumption rate (OCR) (Figure 5A). CD8^+^ T cells from peripheral blood of patients and healthy donors showed a similar pattern following stimulation (Supplemental Figure 4, A and B). The increased glycolysis of RCC CD8^+^ TILs with CD28 costimulation suggested increased glucose uptake. Mass cytometry data, however, indicated that GLUT1 expression was only moderately increased. T cells can express multiple glucose transporters, and consistent with gene expression indicating GLUT3 upregulation, we found GLUT3 to be significantly induced by CD28 costimulation at the protein level, while GLUT1 was not (Figure 5B). CD8^+^ T cells from peripheral blood of patients and healthy donors showed a similar pattern following stimulation (Supplemental Figure 4, A and B).

### CD28 costimulation drives glucose metabolism to rescue CD8^+^ TIL mitochondria and effector functions.

Our data show that CD8^+^ RCC TILs are capable of increasing markers of activation and effector function following activation with CD28 costimulation, and this is associated with increased glycolytic and oxidative activity. The contribution of glucose metabolism to T cell activation and function was next tested. Addition of the glycolytic end product pyruvate to the culture media to bypass glycolysis and provide glucose-derived mitochondrial fuel resulted in an increase in activation markers and improvement in effector function for some patients (Supplemental Figure 5A). Glutamine can also provide a fuel for T cell mitochondrial metabolism, and inhibiting this pathway can lead to increased glycolysis that may promote T cell effector function (11, 12). To test this, we added the glutaminase inhibitor CB839 to RCC TIL culture conditions and found that it enhanced granzyme B production from CD8^+^ RCC TILs in a subset of patient samples (Supplemental Figure 5B). These data suggest that increased glucose metabolism is sufficient to enhance TIL function.

**Figure 6:**
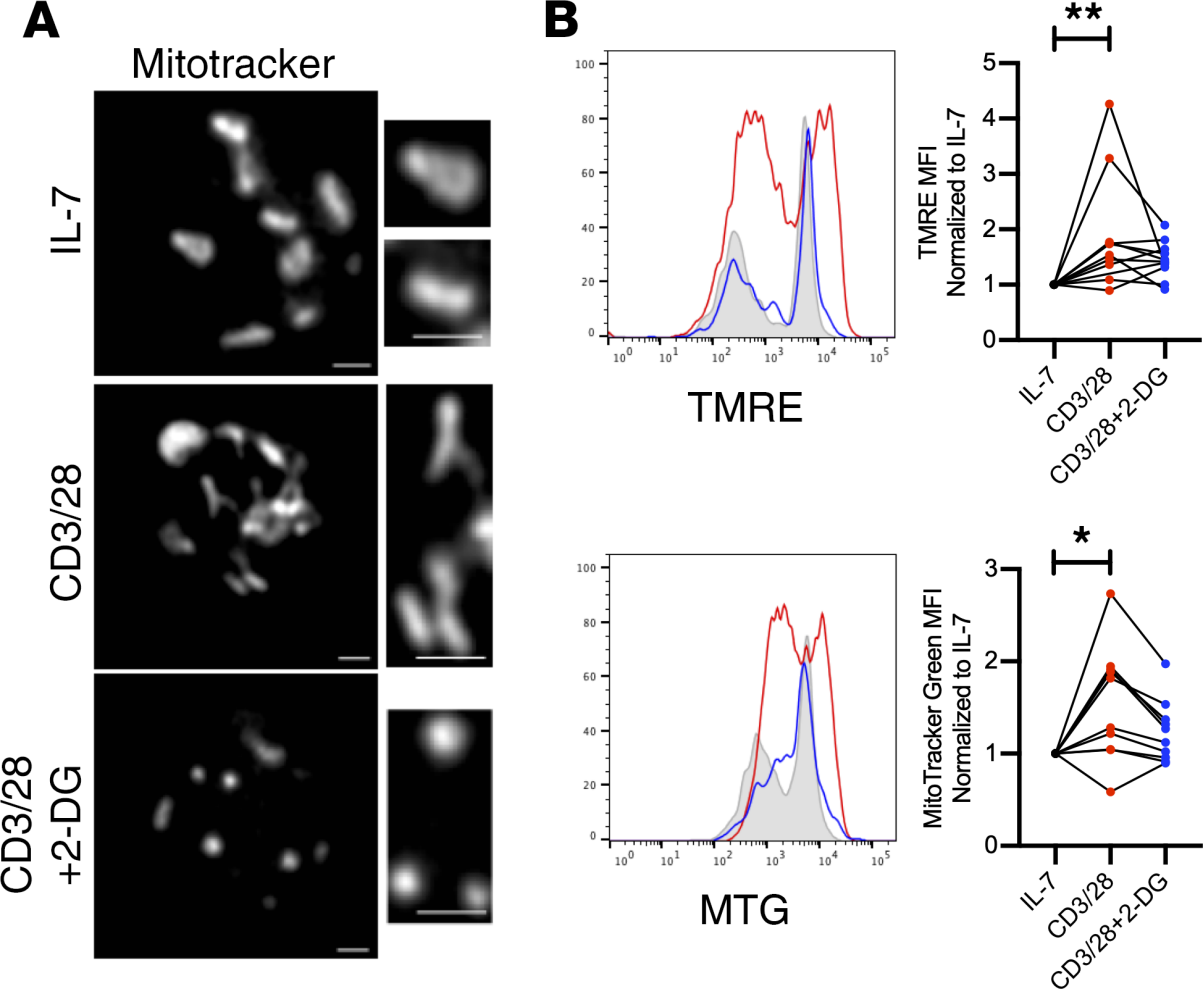
Mitochondrial structure and function are enhanced by CD28 costimulation. (**A**) Mitochondrial structure assessed by immunofluorescence using MitoTracker Deep Red for labeling of mitochondria of cells treated with IL-7, CD3 and CD28 costimulation, or CD3 and CD28 costimulation with the inhibitor 2-DG. Images are representative of cells from *n* = 3 patients. Scale bars: 1 mm. (**B**) Assessment of mitochondrial function by flow cytometry measuring electron membrane potential using TMRE and mitochondrial mass using MitoTracker Green (MTG) in RCC TILs treated with costimulation, or costimulation with 2-DG, with lines connecting individual patient samples for each condition. *n* ≥ 10. *P < 0.05, **P < 0.01 by 1-way ANOVA with Tukey’s post hoc test.

**Figure 7:**
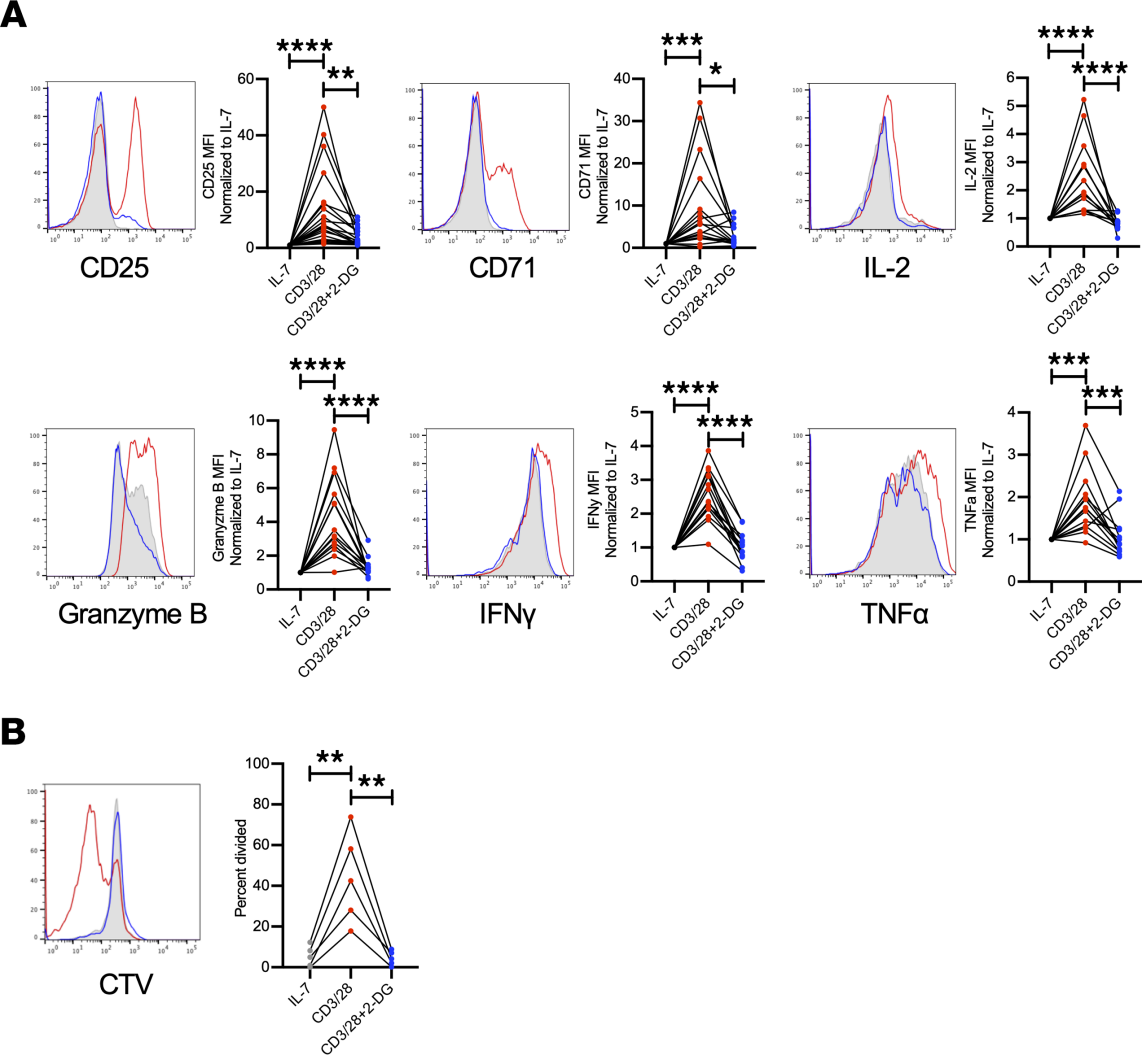
CD28 costimulation increased CD8^+^ RCC TIL activation requires glycolysis. (**A**) Flow cytometry analysis of surface markers of activations (CD25, CD71) and effector function (IL-2, granzyme B, IFN-γ, and TNF-α) following 5 days of RCC TIL coculture, normalized to IL-7 and compared with CD3 with CD28 costimulation or treated with costimulation and 2-DG. *n* ≥ 13. *P < 0.05, **P < 0.01, ***P < 0.001, ****P < 0.0001 by 1-way ANOVA with Tukey’s post hoc test. (**B**) Replication of CD8^+^ RCC TILs assessed following staining with CellTrace Violet and analyzed on day 5 following treatment with either CD3/CD28 costimulation or CD3/CD28 costimulation and 2-DG. *n* = 5. *P < 0.05, **P < 0.01, ***P < 0.001, ****P < 0.0001 by 1-way ANOVA with Tukey’s post hoc test.

The increased oxidative metabolism revealed by gene expression and extracellular flux analyses suggested a role for mitochondrial metabolism in stimulated CD8^+^ RCC TILs. Because CD28 costimulation increased mitochondrial oxygen consumption and exogenous application of pyruvate could restore some TIL functional characteristics, we tested whether CD28 rescue of TIL metabolism and function occurred via mitochondrial fitness, and whether this effect was dependent on glucose metabolism and glycolysis. Mitochondria of resting TILs exposed only to IL-7 were fragmented; however, results of activation with CD28 costimulation suggested mitochondrial fusion (Figure 6A). The role of glucose metabolism and glycolysis in RCC CD8^+^ TIL activation was tested using 2-deoxy-d-glucose (2-DG), a competitive inhibitor that reduces glucose-6-phosphate production from glucose. This reorganization of mitochondrial structure, however, was prevented by treatment by the glycolytic inhibitor 2-DG, and instead the T cells demonstrated further mitochondrial fission. Costimulation with CD28 also partially restored mitochondrial function through increased glycolysis, as mitochondrial potential and mass were increased in a glycolysis-dependent manner in TILs activated with CD28 costimulation (Figure 6B). CD8^+^ T cells from peripheral blood of patients and healthy donors showed a similar pattern following stimulation (Supplemental Figure 6). TIL parameters associated with effector activity were next measured upon activation with costimulation alone or together with 2-DG to inhibit glycolysis. Importantly, the increases in effector markers, cytokine secretion, and proliferation observed in CD28-costimulated RCC CD8^+^ TILs were prevented by inhibition of glycolysis with 2-DG (Figure 7). CD8^+^ T cells from peripheral blood of patients and healthy donors showed a similar pattern following stimulation (Supplemental Figure 7). Together, these data show that RCC CD8^+^ TILs can induce a metabolically active state with improved effector function when provided adequate CD28 costimulation and do so in a glycolysis-dependent manner to maintain mitochondrial metabolism.

## Discussion

All cells require nutrients to complete their function and fate, and the tumor microenvironment may be a particularly hostile metabolic environment. The complexity of cellular interactions, depletion of available nutrients, and potential build-up of waste products may restrict T cell function, adding to the immune-suppressive character of tumors. Blocking the negative regulators of PD-1 and CTLA4 that impair CD28 signaling to release inhibition of T cells can allow for antitumor activity. Therapeutically, inhibiting the negative T cell regulators CTLA4 and PD-1 has resulted in dramatic responses in patients with cancer who previously had grave prognosis. The biology that allows for a patient to respond to such therapy, however, is not fully understood. Kidney cancer has long been known to be resistant to traditional cytotoxic chemotherapy but can be responsive to immunotherapy (37). However, only 25% of RCC patients respond to treatment with single-agent monoclonal antibodies targeting PD-1, and approximately 40% respond with combined blockade of CTLA4 and PD-1 (27, 28). Given the successes of approaches to block the inhibitory receptors PD-1 and CTLA4 that act in part through inhibition of CD28 signaling, it is now important to better understand the role of costimulatory signals in TIL function to further increase the efficacy of these treatments.

The biologic driver of clear cell RCC starts with the mutations in the *VHL* gene that lead to stabilization of HIF1A and higher rates of aerobic glycolysis than in normal tissue (25, 26). This may create an environment of nutrient competition for T cells, as well as buildup of waste products that may impair T cells (3, 38). T cells in the tumor microenvironment rely on adaptation of different metabolic pathways to meet the energy demands necessary for cell division and effector function (39). It is clear that classical T cell activation is dependent upon upregulation of glucose uptake and access to other essential nutrients. Glucose uptake through the glucose transporter GLUT1 (SLC2A1) increases dramatically after T cell activation in settings of infection and inflammation (40). In T cell activation following T cell receptor engagement and costimulation with CD28, restricting glucose uptake can be limiting to T cell effector function (34).

The tumor microenvironment may also change T cell metabolism through signaling by checkpoint molecules. For example, PD-1 signaling suppresses T cell metabolic reprogramming by inhibiting glycolysis and instead promoting lipolysis and FAO (15). Recently, in vitro models showed that PD-L1 inhibition stopped de novo nucleoside phosphate synthesis (16). CTLA4 can inhibit CD28 signaling and has also been shown to impair metabolic reprogramming and induction of glycolysis. Mitochondria and mitochondrial function are integrally important for the activity of T cells, and the tumor microenvironment may limit glycolysis while also repressing T cell mitochondrial biogenesis.

Our results show that while CD8^+^ RCC TIL gene expression exhibits classical markers of chronic stimulation as well as enrichment of metabolic pathways that include FAO, glycolysis, and cholesterol homeostasis, a large portion of cells could be stimulated to reprogram metabolism and induce effector functions. Single-cell proteomic analysis using mass cytometry shows that CD8^+^ cells clustered into unique subsets based on surface marker expression that were similar across patients for each sample of origin, including PBMCs, RCC tumor, and adjacent kidney tissue. While in some cases healthy donor PBMCs were examined due to limitations of tissue availability and patient safety, we found these to be similar to those from patient peripheral blood. Specifically, CD8^+^ T cells in tumor as compared with adjacent kidney tissue or matched peripheral blood had subsets with high expression of HLA-DR and PD-1, in agreement with previous publications (30). CyTOF analysis showed that CD8^+^PD-1^+^ cells in tumor had decreased expression of ATP5a, cytochrome *c*, and GLUD1, suggesting that these T cells decreased utilization of the electron transport chain for energy production. This lack of active metabolism through oxidative phosphorylation suggests that CD8^+^ TILs from RCC are in a chronically stimulated environment where overall energy requirements are low as compared with surveilling CD8^+^ T cells in the periphery (41).

Our prior work showed that RCC CD8^+^ TILs have metabolic defects, and we tested here whether the costimulation known in classical regulation of T cells to increase glycolysis could overcome this metabolic defect seen in patient TILs (5, 18). Costimulation using CD28 increased markers of activation and effector function in patient RCC TILs in a pattern similar to that observed in prior studies with preclinical and patient samples of other tumor types showing that costimulation using CD28 or 4-1BB can increase antitumor activity (7, 42, 43). To understand the mechanism behind CD28 costimulation resulting in increased CD8^+^ TIL activity, single-cell gene expression analysis of RCC patient TILs treated with varying stimulation conditions was compared with peripheral blood CD8^+^ cells. Peripheral blood cells showed marked differences between control and all stimulation conditions, while CD8^+^ TILs at baseline exhibited a chronically stimulated state that only changed with CD28 costimulation. At the single-cell level, costimulation shifted the percentage of cells from a baseline resting state into 2 primary branches: one that was enriched in IL-2 signaling and glycolysis, and another that exhibited pathways of glycolysis, oxidative phosphorylation, and Myc signaling. This bioenergetic switch is consistent with known Myc regulation of metabolic reprogramming during T cell activation (6). At the protein level as measured by mass cytometry, changes in the UMAP populations of RCC CD8^+^ TILs required CD28 costimulation, while peripheral blood CD8^+^ T cell changes were seen with CD3 alone. These cells exhibited increased glycolytic flux, as measured by Seahorse, and had corresponding increases in transcriptional expression of the transmembrane glucose transporter GLUT3. Increased GLUT3 isoform is consistent with previously published data showing an increase in various glucose transporters, including GLUT1, GLUT3, and GLUT6, in T cells following activation (44, 45).

The enhancement in CD8^+^ TIL activation with CD28 costimulation was blocked following the addition of the glycolytic inhibitor 2-DG, indicating that the breakdown of glucose was necessary for downstream metabolic pathways serving the energetic needs of activated CD8^+^ RCC TILs. Similarly, the ability of CD8^+^ TILs to expand was inhibited with the addition of 2-DG. The use of glucose in CD8^+^ RCC TIL activity after CD28 costimulation is consistent with other data in the field finding that phosphoenolpyruvate is necessary for maximal Ca/NFAT signaling to improve antitumor response (46). While our results support that CD28-mediated costimulation of RCC CD8^+^ TILs coincides with increased glycolysis that could provide energy for pentose phosphate pathway or nucleotide synthesis, at least some glucose may aid in increasing mitochondrial fitness with enhanced mitochondrial membrane potential, mass, and structure. These data complement other findings showing that improved mitochondrial fitness can enhance immunotherapy by demonstrating that glucose metabolism can support TIL mitochondria (15, 19, 47, 48).

Efforts to increase the proportion of patients in which checkpoint immunotherapies can be effective will rely on improved understanding of TIL biology and metabolism. The CD28 signaling pathway can play a critical role in T cell metabolism, and our findings show it can also increase the metabolism and function of RCC CD8^+^ TILs. CTLA4 blockade immunotherapy acts in part by allowing CD28 to associate with the ligands CD80 and CD86. Improved glycolysis and mitochondrial fitness through CD28 and other similar costimulatory pathways now provide a new opportunity to enhance TIL function and checkpoint immunotherapies.

## Methods

### Sample collection and processing.

Fresh malignant tissue was surgically removed from 44 patients with clear cell RCC (Supplemental Table 1). In some cases, patient peripheral blood and adjacent healthy kidney tissue was obtained. Patients with confirmed histology of renal oncocytoma, papillary RCC, or chromophobe RCC were excluded from the study. Tissues were processed by mechanical dissociation, followed by enzymatic digestion in deoxyribonuclease I (MilliporeSigma, D5025) and collagenase (MilliporeSigma, C2674). Single-cell suspension was obtained after passage through a 70 μM strainer and subsequent red blood cell lysis (18). Single-cell suspensions were cocultured for 5 days in RPMI 1640 complete supplemented with 10% FBS, 1% glutamine, 1% penicillin/streptomycin, 1% HEPES, and 0.1% 2-mercaptoethanol (BME) before the analysis described below. PBMCs were isolated by density gradient centrifugation using Ficoll-Paque (GE Healthcare, 17144002) in SepMate-50 tubes (STEMCELL Technologies, 85450).

### T cell enrichment and stimulation.

PBMCs and single-cell suspensions of RCC tumor or adjacent kidney tissue were cultured for 5 days in RPMI 1640 complete at 37°C, 5% carbon dioxide, 95% relative humidity for maintenance in 10 nM IL-7, anti-CD3 at 1:1000 μL (clone UCHT1; Invitrogen, 16-0038-85) and/or anti-CD28 at 1:1000 μL (clone CD28.2; Invitrogen, 16-0289-81). 2-DG (MilliporeSigma, D6134) was used at a 10 mM concentration with costimulation. For Seahorse and microscopy experiments, CD8^+^ T cells were isolated from tumor single-cell suspensions using CD8 microbeads (Miltenyi Biotec, 130-045-201).

### Fluorescence cytometry.

Expression of T cell surface markers and intracellular targets was measured by fluorescence cytometry (MACSQuant, Miltenyi Biotec) and analyzed by FlowJo version 10.3 and GraphPad Prism version 8.1.0 software. See Supplemental Table 2 for antibody information. For intracellular cytokine stains, cells were restimulated using 0.1 μg/mL PMA (MilliporeSigma, P8139) and 1.5 mg/mL ionomycin (MilliporeSigma, I0634) in the presence of GolgiPlug at a ratio of 1:500 μL (BD Biosciences, 555029) for 4 hours, then fixed and stained for intracellular cytokines using a Fixation/Permeabilization kit (BD Biosciences, 554714). Cell proliferation was assessed by staining PBMCs or single-cell suspensions with CellTrace Violet proliferative dye at 5 μM (Invitrogen, c34557).

### Assessment of mitochondrial morphology and detection of ROS.

Mitochondrial membrane potential was measured using TMRE (0.2 μM; Invitrogen, T-669) according to the manufacturer’s protocols. Mitochondrial mass was assessed using MitoTracker Green (0.2 μM; Invitrogen, M7514).

### Mass cytometry.

Metal-tagged antibodies were purchased from Fluidigm. Cell labeling and mass cytometry analysis were performed as previously described (49, 50). Briefly, cells were incubated with a viability reagent (Cell ID Intercalator-Rh; Fluidigm), per the product literature. Then cells were washed in PBS without calcium or magnesium (Gibco, Thermo Fisher Scientific) containing 1% BSA (Thermo Fisher Scientific) and stained in 50 μL PBS and BSA 1%–containing antibody cocktail for extracellular targets. Cells were stained for 30 minutes at room temperature using the antibodies listed in Supplemental Table 3. Cells were washed in PBS and BSA 1% and then fixed with 1.6% paraformaldehyde (Electron Microscopy Sciences). Cells were washed once in PBS and permeabilized by resuspension in ice-cold methanol. After incubation overnight at −20°C, cells were washed with PBS and BSA 1% and stained in 50 μL PBS and BSA 1%–containing antibody cocktail for intracellular targets. Cells were washed in PBS and BSA 1%, then washed with PBS and stained with an iridium DNA intercalator (Fluidigm) for 20 minutes at room temperature. Finally, cells were washed with PBS and with diH_2_O before being resuspended in 1× EQ Four Element Calibration Beads (Fluidigm) and collected on a Helios mass cytometer (Fluidigm) at the Vanderbilt Flow Cytometry Shared Resource Center. Events were normalized as previously described (51).

### Mass cytometry data analysis.

After normalization, data were scaled with an ArcSinh transformation, with an appropriate cofactor set for each channel following standard procedures for fluorescence and mass cytometry data (52). Live single immune cells were identified as CD45^+^. A UMAP analysis, from the uwot package in R, was performed using 15 of the measured markers on all live single immune cells from each of the 15 samples (2,306,743 total cells). The resulting common, 2-dimensional embedding of the data was used for visualization and selection of CD8^+^ T cells for further downstream analysis. t-SNE analysis was performed using 12 of the measured markers on an equal number of the CD8^+^ T cells extracted from the UMAP from 12 of the 15 samples (20,376 total cells; 1,698 cells selected randomly from 12 samples). MEM was then used to quantitatively describe the phenotype of those CD8^+^ T cells (for patients 166, 167, 198) within a given tissue type, as compared with all other cells in the other tissue types. MEM calculates the median and interquartile range, and calculates a relative enrichment score on a –10 to +10 scale (53). Positive MEM scores indicate that a cluster was specifically enriched for a protein feature, and negative scores signify that a cluster specifically lacked a protein feature, in relation to all other cells. EMD was also calculated for each sample compared with every other sample in the data set for comparisons of differences between t-SNE maps.

For the stimulation experiment, mass cytometry data analysis remained largely similar. However, UMAP was used on the selected CD8^+^ T cells for further downstream analysis instead of t-SNE. The UMAP analysis was performed using 24 of the measured markers on all CD8^+^ T cells (98,620 total cells) from the healthy control and tumor sample for all conditions. Once again, EMD was calculated to quantify differences in the low-dimensional projections of the data as well as MEM scores to quantify phenotypic signatures in the samples.

### Extracellular flux analyses (Seahorse).

Experiments were carried out on an Agilent Seahorse XF96 bioanalyzer. CD8^+^ T cells were isolated as above and spun onto XF96 Cell-Tak–coated (Corning, 354240) plates (Agilent, 101085-004) and rested in Seahorse XF RPMI 1640 media (Agilent, 103576-100) supplemented with 1% l-glutamine. A Seahorse XF glycolysis stress test kit was used (Agilent, 103020-100) by sequentially adding glucose (10 mM glucose), oligomycin (1 μM), and 2-DG (20 mM). Glycolytic function was measured by ECAR (mpH/min) and OCR (pmol/min).

### Microscopy.

For immunofluorescence microscopy, CD8^+^ T cells were isolated as described above and incubated with 100 nM MitoTracker Deep Red (Invitrogen, M22426) for 15 minutes, fixed with 4% PFA (Electron Microscopy Sciences) in PBS, stained with 300 nM DAPI, and mounted with a Wescor Cytopro cytocentrifuge, then ProLong Gold Antifade reagent (Thermo Fisher Scientific). Images were acquired on a Zeiss LSM880 confocal microscope with Airyscan (Vanderbilt University Cell Imaging Shared Resource) for super-resolution imaging using a 63×/1.40 NA Plan-Apochromat oil objective and 405-, 633-nm lasers and default settings for pinhole (~2–3 airy units) and filters. Images were processed using FIJI (http://fiji.sc) and ZEN (Zeiss) software (using default Airyscan settings). To process mitochondrial images and remove nonspecific MitoTracker staining, nuclear MitoTracker signal was quantified, then 2 SDs from the mean intensity was subtracted from all slices of MitoTracker *Z*-stack. Images presented are single *Z*-slices, and Supplemental Videos are presented as serial *Z*-slices through the volume of the cell.

### Staining, sorting, and RNA extraction and sequencing.

The frozen tumor and PBMC suspensions were thawed using AIM V media (10% FBS), washed twice in media, and counted. The cells were washed twice in PBS and stained with a viability dye. The cells were then washed twice in staining buffer (RPMI 1640 with no phenol red, 4% FBS) and Fc blocked with mouse immunoglobulin (MilliporeSigma) for 10 minutes, followed by surface staining for 30 minutes on ice using the following: CD19-BB515, CD8-PE, CD14/CD16-PerCPCy5.5, CD45-BV421, CD27-APC, FVS780. The cells were washed twice and resuspended in sort buffer (RPMI 1640 with no phenol red, 4% FBS, 25 mM HEPES). CD8^+^ T cells (and B cells) were sorted from each sample on a Jazz cell sorter using a 100 μm nozzle into complete media AIM V (10% FBS). The sorted cells were washed twice in PBS, resuspended in 75 μL QIAGEN RLT buffer, flash frozen on dry ice, and stored at –80°C until RNA extraction.

Samples were thawed on ice and vortexed for 1 minute. RNA was extracted using a QIAGEN RNeasy Micro kit (catalog 74004) with on-column DNA digestion per the manufacturer’s instructions. RNA quality was assessed with an Agilent TapeStation RNA high-sensitivity kit, with libraries created using Clontech SMARTer Stranded RNA-Seq kit (catalog 634836). Libraries were sequenced by the UNC High Throughput Sequencing Facility (HTSF) using a HiSeq 4000 instrument (Illumina).

### RNA-Seq data analysis.

Raw read quality was assessed using FastQC (v0.11.5. https://www.bioinformatics.babraham.ac.uk/projects/fastqc). Salmon (v0.14.0, https://combine-lab.github.io/salmon/) was used to quantify transcript expression reads using annotation from UCSC Known Gene transcriptome (http://hgdownload.cse.ucsc.edu/goldenPath/hg38/database/knownGeneMrna.txt.gz) under quant mode with default parameters (transcript index was generated with *k* = 31). Transcript expression was summarized at the gene level and imported into R using txImport (https://bioconductor.org/packages/release/bioc/html/tximport.html). Then the differentially expressed genes were called using edgeR (v2.26.5) with Benjamini-Hochberg–adjusted (BH-adjusted) *P* value less than 0.01. The GSEA analysis was applied to fold change ranked gene list using clusterProfiler (v3.12.0) on hallmark gene sets. CIBERSORT analysis was performed using default parameters using signature genes of T cell subsets from non–small cell lung cancer (31). Heat map was generated using pheatmap (v1.0.12).

### Single-cell RNA-Seq data methods and analysis.

For single-cell RNA-Seq, PBMCs and single-cell tumor suspensions were cultured as above and on day 5, viable cells were isolated using flow sorting based on propidium iodide, and each sample was processed for single-cell 5′ RNA sequencing using the 10× Genomics Chromium System. Libraries were prepared using P/N 1000006, 1000080, and 1000020 following the manufacturer’s protocol. A quality control assessment was completed for each library, including fluorometric quantitation, library profile analysis using the Agilent Bioanalyzer, and quantitative PCR (qPCR). The libraries were sequenced using the NovaSeq 6000 with 150 bp paired-end reads. RTA (version 2.4.11; Illumina) was used for base calling, and analysis was completed as follows.

CellRanger software (v3.0.2, https://github.com/10XGenomics/cellranger) was used with default parameters for library demultiplexing, fastq file generation, read alignment, and unique molecular identifier (UMI) quantification to generate the gene expression matrix. Aggregated gene expression matrices containing numbers of UMIs per cell per gene were filtered to retain cells with at least 100 genes detected and less than 10% of total UMIs originating from mitochondrial RNA. Genes detected in more than 3 cells were retained for the following analysis. Dimension reduction (principal component analysis [PCA], UMAP) and clustering were applied to the filtered matrix using Seurat (v3.0) with default parameters, except the top 20 dimensions of PCA were used for UMAP. Cell trajectory modeling was performed using either phateR (v0.4.1; gamma = 0, knn = 10, t = 80) or monocle (v2.12.0; method = “DDRTree”); the trajectory branches (states) were assigned using monocle (v2.12.0). AUC scores of hallmark pathways for each cell were calculated using the R package AUCell. Data visualization was done using the respective analysis tools or custom scripts using ggplot2 (R package). Data sets have been deposited in BioProject (accession PRJNA616283) and are publicly available in the NCBI’s Gene Expression Omnibus database (GEO GSE151669).

### Quantification and statistics.

Statistical analyses were performed with GraphPad Prism software version 8.1.0. For data involving 2 groups, analysis was performed using Student’s *t* test. For data involving 3 or more groups, analysis was performed using 1-way ANOVA with Tukey’s post hoc multiple-comparisons test.

### Study approval.

All donor samples were collected and studies conducted in accordance with the Declaration of Helsinki principles under a protocol (no. 151549) approved by the Vanderbilt University Medical Center IRB. Written informed consent was received from all patients and healthy donor participants before inclusion in the study.

## Author contributions

KEB designed and performed the experiments, analyzed the data, and wrote the manuscript. WKR and JCR designed experiments and wrote the manuscript. KY, RH, and KC performed experiments and processed RCC tissue. XY and CCS analyzed RNA-Seq data. DCCH and CER performed mass cytometry antibody panel creation and antibody staining and cytometry experiments. SB analyzed single-cell mass cytometry data. FMM performed immunofluorescence experiments. BGV, JMI, WKR, and JCR evaluated the data.

## Supplementary Material

Supplemental data

Supplemental Table 1

Supplemental Table 2

Supplemental Table 3

## Version 1 08/20/2020

Electronic publication
