# Supplementary material for: CD28 costimulation drives tumor-infiltrating T cell glycolysis to promote inflammation
Source: JCI Insight. 2020 Aug 20;5(16):e138729. doi: 10.1172/jci.insight.138729 (PMC7455120; doi:10.1172/jci.insight.138729)
Supplement: Supplemental data [file jciinsight-5-138729-s087.pdf]

SUPPLEMENTAL FIGURE LEGENDS

Supplemental Figure 1. Gene expression analysis of CD8 cells from RCC TIL and patient blood

A) CIBERSORT estimation of cell subsets abundances in blood CD8 t cell (white) vs RCC TIL (grey). One asterisk equivalent to 0.05 and two asterisks equivalent to 0.005 as calculated by paired student's t-test.

B) Expression of immune checkpoint genes shown as a log fold change from CD8 of RCC TIL compared to patient peripheral blood CD8+ T cell. Red lines indicate P=0.01, p values were calculated by quasi-likelihood method with edgeR.

C) GSEA plot for enriched hallmark gene sets and heatmap depiction of differentially expressed genes (BH adjusted p value < 0.05) in the enriched hallmark gene sets.

D) Flow cytometry analysis of CD28 expression showing mean fluorescence intensity and percentage of total CD8 from TIL and patient peripheral blood.

Supplemental Figure 2. Healthy donor CD8 show increased markers of activation (CD25 and CD71) and effector function (Granzyme B) with co-stimulation by CD28.

Single cell suspensions were cultured with IL7 (grey) to maintain homeostasis, CD3 alone (black) for TCR engagement, or CD3 and CD28 co-stimulation (red) for 5 days before perform flow cytometry. CD8 cells assessed for markers of activation using CD25 and CD71 as well as effector function using Granzyme B in CD8 (n>=9). * p < 0.05, ** p <0.01, *** p < 0.001, **** p<0.0001 by 1-way ANOVA with Tukey post hoc test.

Supplemental Figure 3. Pathway activities of cells visualized by PHATE.

Supplemental Figure 4. Metabolic activation by CD28 in patient and healthy donor peripheral blood CD8 T cells

A) Glycolytic stress test performed showing representative ECAR and OCR from patient CD8 blood and following IL7 (grey), CD3 alone (black), CD3 with CD28 co-stimulation (red). Flow cytometry analysis showing MFI of GLUT1 (N>=5) and GLUT3 (N>=8) normalized to IL7 and comparing CD3 with CD28 co-stimulation (red). * $p < 0.05$, ** $p < 0.001$ by 1-way ANOVA with Tukey post hoc test.
B) Glycolytic stress test performed showing representative ECAR and OCR from healthy donor blood and following IL7 (grey), CD3 alone (black), CD3 with CD28 co-stimulation (red). Flow cytometry analysis showing MFI of GLUT1 and GLUT3 normalized to IL7 and comparing CD3 with CD28 co-stimulation (red). N>=4

Supplemental Figure 5. Metabolic manipulation of RCC CD8 TIL can increase activity.

A) Treating RCC TIL co-culture with IL7 (grey), CD3 with CD28 co-stimulation, or CD3, CD28, and 20 mM pyruvate supplementation shows increased activation and effector function with pyruvate addition (n=3/6).
B) Treating RCC TIL co-culture with IL7 (grey), CD3 with CD28 co-stimulation (black), or CD3 and CD28 co-stimulation with CB839 inhibitor of glutaminolysis can increase Granzyme B in subset of RCC TIL (n=2/10).

Supplemental Figure 6. CD28 increased membrane potential and mitochondrial mass in patient (A) and healthy donor (B) peripheral blood in CD8 T cells

Supplemental Figure 7. CD28 co-stimulation increased CD8 activation and proliferation requires glycolysis in patient (A) and healthy donor (B) peripheral blood

SUPPLEMENTAL TABLES

Supplemental Table 1. Patient characteristics

Supplemental Table 2. Antibodies for fluorescent flow cytometry

Supplemental Table 3. Mass cytometry antibodies.

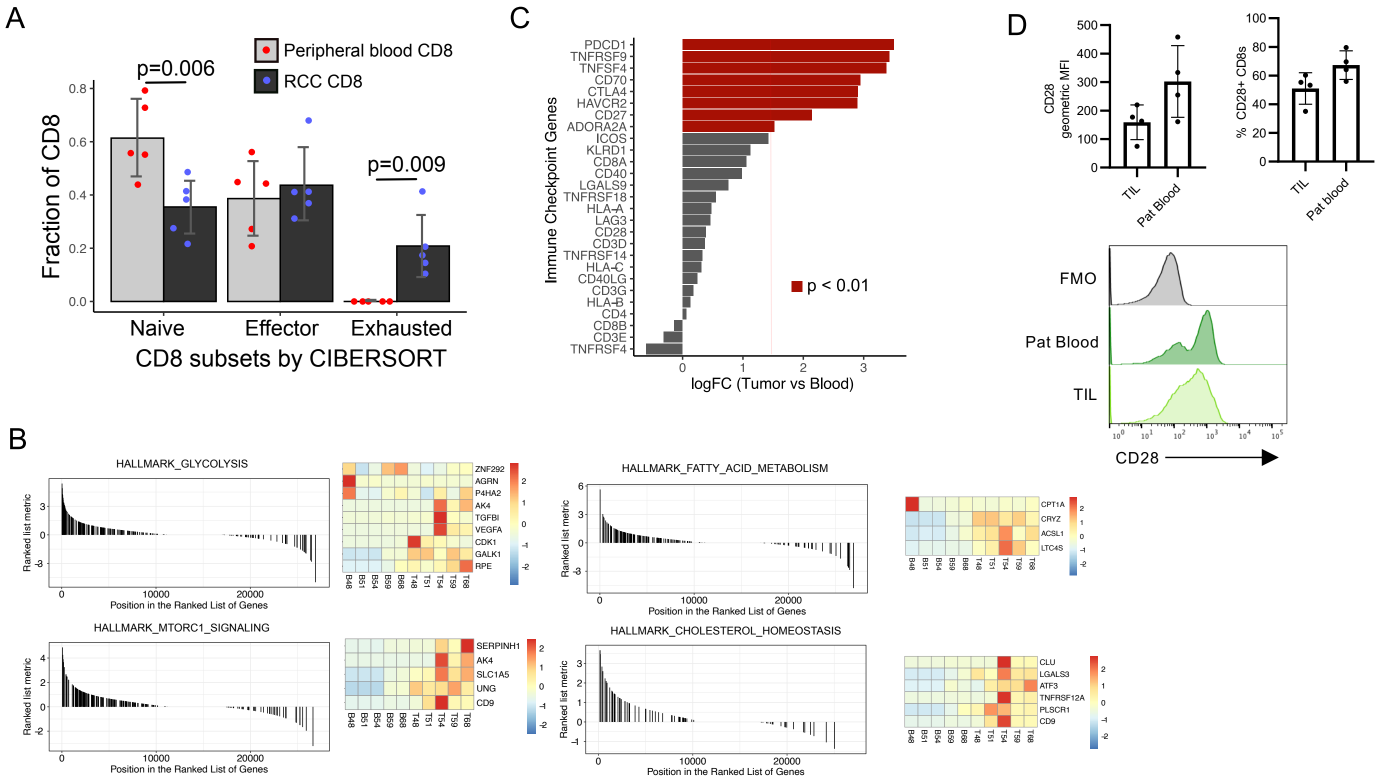

Supplemental Figure 1

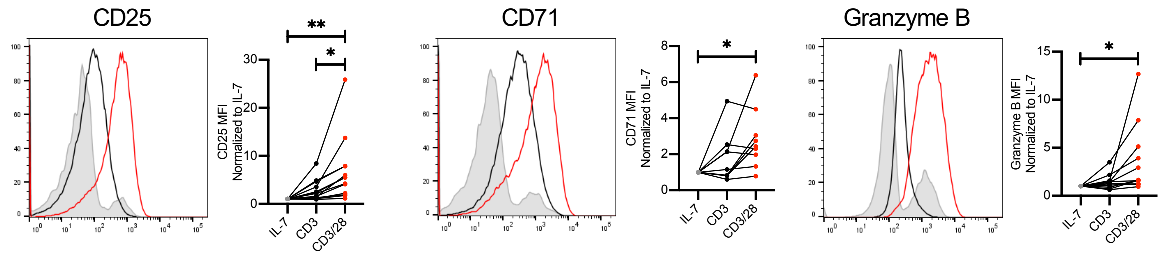

Supplemental Figure 2

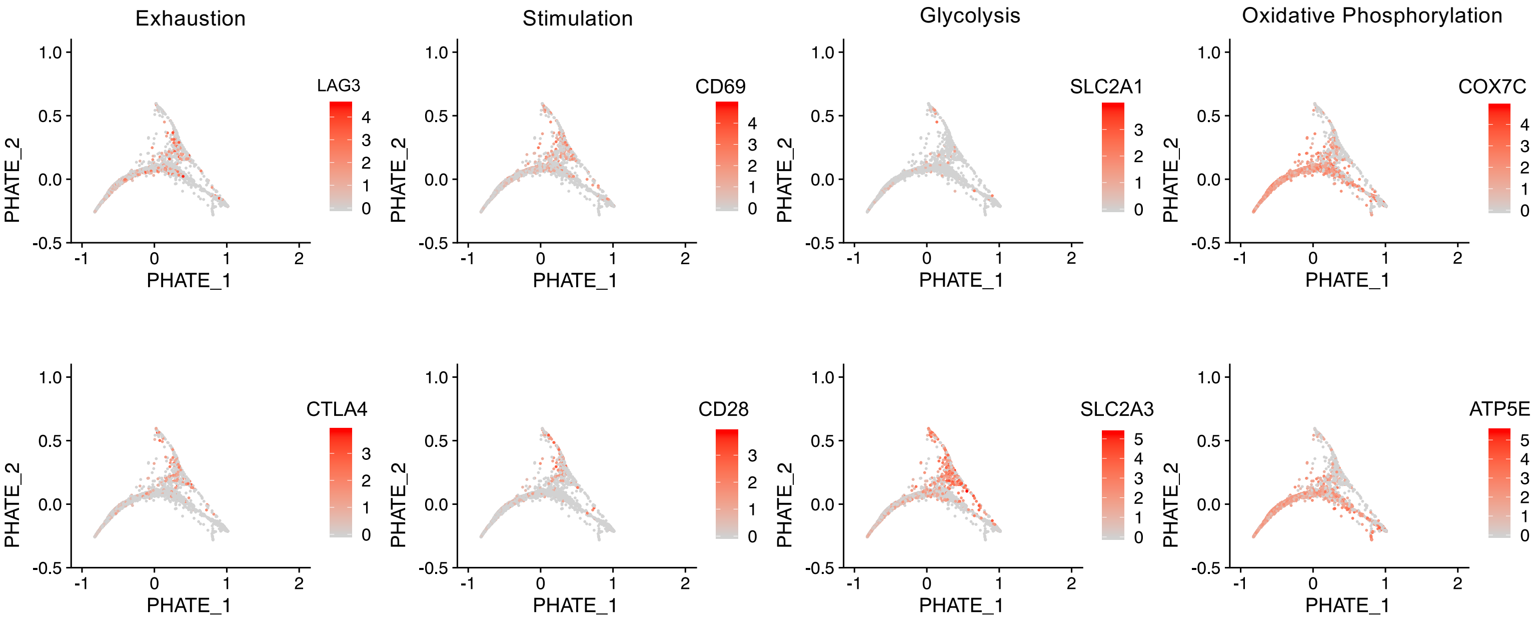

Supplemental Figure 3

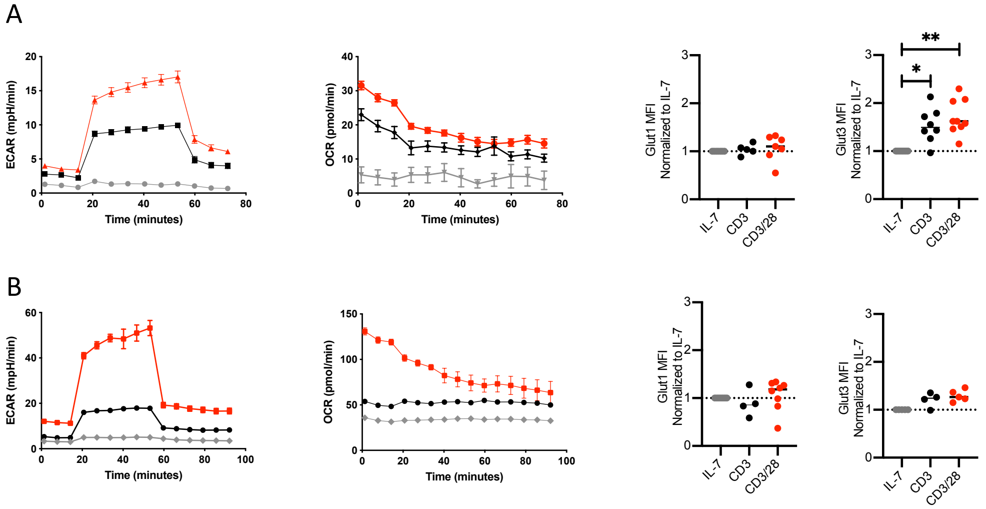

Supplemental Figure 4

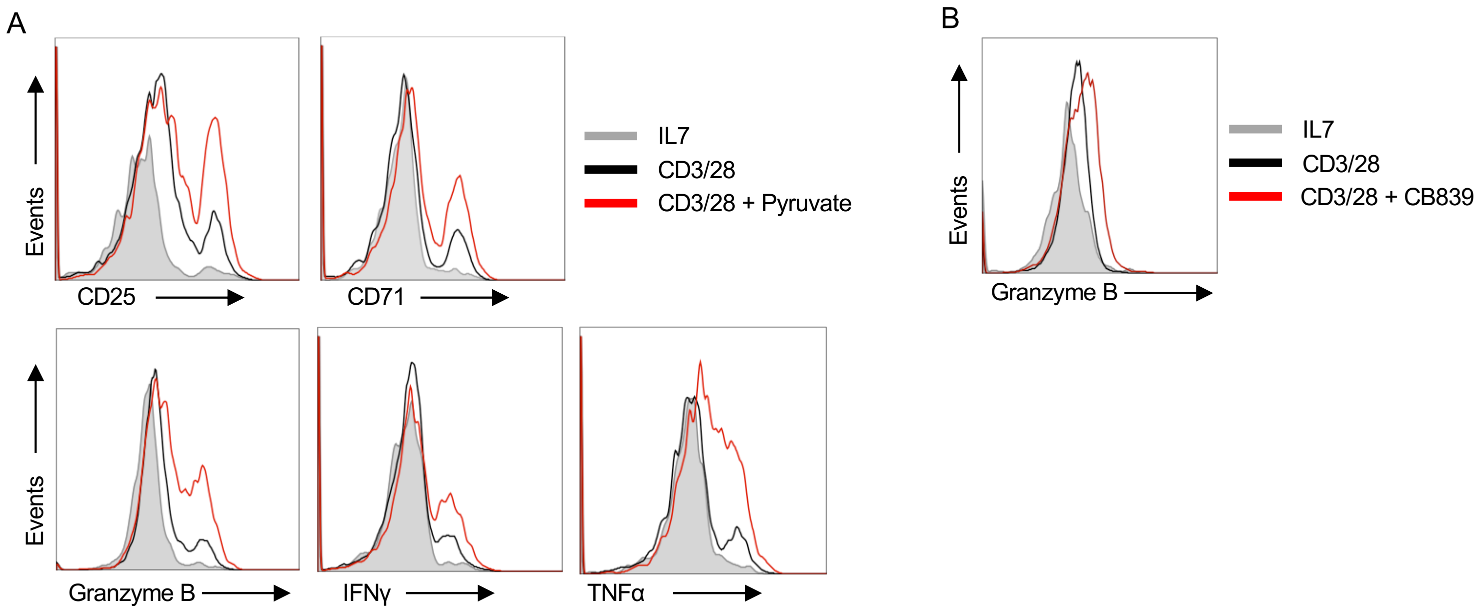

Supplemental Figure 5

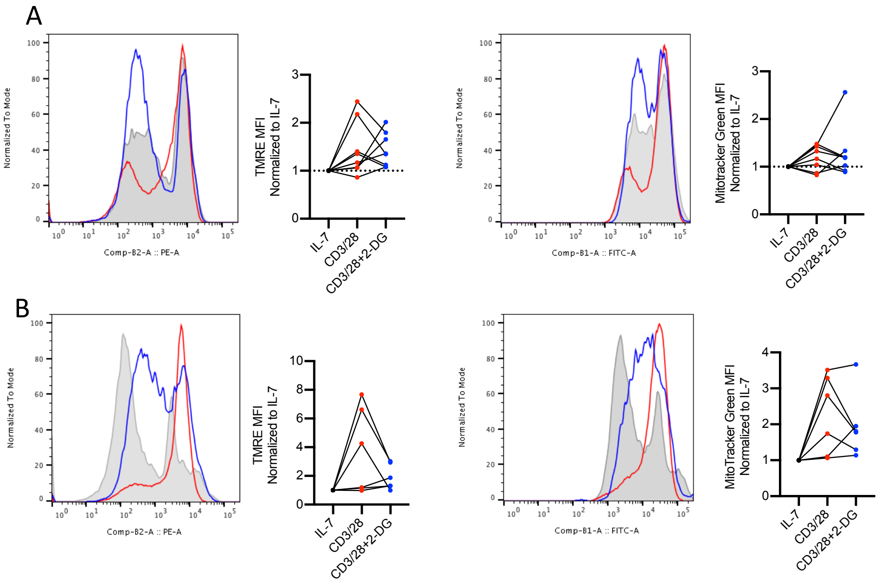

Supplemental Figure 6

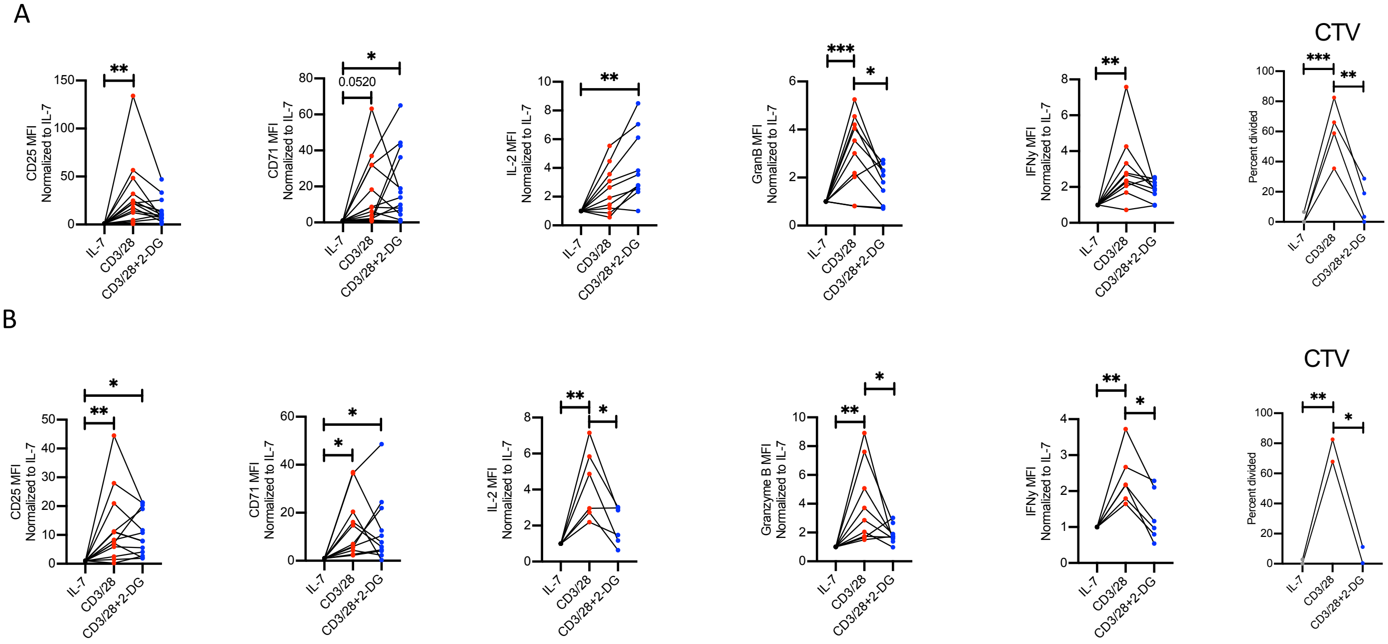

Supplemental Figure 7

Supplemental Table 1. Patient characteristics

Lab ID	Disease	Subtype	Grade	Age	Sex	Race
197	Renal Cell Carcinoma	Clear Cell	2	58	female	white
198	Renal Cell Carcinoma	Clear Cell	2	60	female	white
213	Renal Cell Carcinoma	Clear Cell	4	59	male	white
215	Renal Cell Carcinoma	Clear Cell	3	55	male	white
220	Renal Cell Carcinoma	Clear Cell	3	44	male	white
227	Renal Cell Carcinoma	Clear Cell	3	62	male	white
228	Renal Cell Carcinoma	Clear Cell	2	67	male	hispanic
240	Renal Cell Carcinoma	Clear Cell	2	62	male	black
248	Renal Cell Carcinoma	Clear Cell	2	66	female	white
251	Renal Cell Carcinoma	Clear Cell	3	67	male	white
252	Renal Cell Carcinoma	Clear Cell	2	44	female	white
254	Renal Cell Carcinoma	Clear Cell	3	48	male	white
257	Renal Cell Carcinoma	Clear Cell	2	43	male	white
263	Renal Cell Carcinoma	Clear Cell	2	63	male	white
269	Renal Cell Carcinoma	Clear Cell	3	67	male	white
271	Renal Cell Carcinoma	Clear Cell	3	65	male	black
274	Renal Cell Carcinoma	Clear Cell	2	68	male	white
277	Renal Cell Carcinoma	Clear Cell	2	47	male	white
283	Renal Cell Carcinoma	Clear Cell	2	55	male	white
287	Renal Cell Carcinoma	Clear Cell	2	74	female	white
292	Renal Cell Carcinoma	Clear Cell	2	71	female	white
293	Renal Cell Carcinoma	Clear Cell	3	34	female	white
295	Renal Cell Carcinoma	Clear Cell	1	65	female	white
298	Renal Cell Carcinoma	Clear Cell	3	68	male	white
301	Renal Cell Carcinoma	Clear Cell	2	66	female	white
305	Renal Cell Carcinoma	Clear Cell	2	44	female	white
307	Renal Cell Carcinoma	Clear Cell	3	52	male	white
309	Renal Cell Carcinoma	Clear Cell	4	76	female	white
314	Renal Cell Carcinoma	Clear Cell	2	63	female	white
315	Renal Cell Carcinoma	Clear Cell	2	68	male	white
317	Renal Cell Carcinoma	Clear Cell	2	57	male	white
322	Renal Cell Carcinoma	Clear Cell	3	72	male	white
323	Renal Cell Carcinoma	Clear Cell	4	84	male	white
324	Renal Cell Carcinoma	Clear Cell	3	48	male	white
325	Renal Cell Carcinoma	Clear Cell	3	56	female	white
326	Renal Cell Carcinoma	Clear Cell	2	64	male	white
328	Renal Cell Carcinoma	Clear Cell	1	62	male	white
333	Renal Cell Carcinoma	Clear Cell	3	43	male	white
334	Renal Cell Carcinoma	Clear Cell	2	65	male	white
337	Renal Cell Carcinoma	Clear Cell	3	58	male	white
339	Renal Cell Carcinoma	Clear Cell	3	66	male	white
341	Renal Cell Carcinoma	Clear Cell	3	40	male	white
342	Renal Cell Carcinoma	Clear Cell	3	51	female	white
345	Renal Cell Carcinoma	Clear Cell	2	45	male	white

Supplemental Table 2. Antibodies for fluorescent flow cytometry

Antigen	Fluorochrome	Clone	Company	Catalog #	Dilution
CD8	eFluor 450	SK1	Invitrogen	48-0087-42	1:400
CD8	PE	RPA-T8	Invitrogen	12-0088-42	1:400
CD25	PerCP-Cy5.5	BC96	Invitrogen	45-0259-42	1:400
CD71	PE	OKT9	Invitrogen	12-0719-42	1:400
CD71	APC	OKT9	Invitrogen	17-0719-42	1:400
Granzyme B	FITC	REA226	Miltenyi	130-118-341	1:200
Glut1	PE	202915	R&D Systems	FAB1418P	1:100
Glut3	FITC	polyclonal	abcam	136180	1:50
IFNy	PECy7	4S.B3	Invitrogen	25-7319-82	1:100
TNFa	eFluor 450	MAb11	Invitrogen	48-7349-42	1:100
IL-2	APC	MQ1-17H12	Invitrogen	17-7029-82	1:100
CD28	APC	CD28.2	Invitrogen	17-0289-42	1:50

Supplemental Table 3. Mass cytometry antibodies.

Target	Provider	Product #	Clone
Glut1	Novus	NB110-39113	Poly
HK II	Abcam	ab131196	1E8-H3-F11
Grim19	Abcam	ab110240	6E1BH7
ATP5a	Abcam	ab110273	7H10BD4F9
CPT1a	Abcam	ab128568	8F6AE9
CytoC	BD Pharmingen	556432	6H2.B4
Glud1	Abcam	ab34786	Poly
CD45	Fluidigm	3141009B	HI30
CD19	Fluidigm	3142001B	HIB19
CD5	Fluidigm	3143007B	UCHT2
CD4	Fluidigm	3145001B	RPA-T4
CD8a	Fluidigm	3146001B	RPA-T8
CD134/OX40	Fluidigm	3150023B	ACT35
CD62L	Fluidigm	3153004B	DREG-56
CD3	Fluidigm	3154003B	UCHT1
CD27	Fluidigm	3155001B	L128
CD69	Fluidigm	3162001B	FN50
CD44	Fluidigm	3166001B	BJ18
CD25	Fluidigm	3169003B	2A3
HLA-DR	Fluidigm	3170013B	L243
Ki-67	Fluidigm	3172024B	B56
Granzyme B	Fluidigm	3173006B	GB11
CD279/PD-1	Fluidigm	3174020B	EH12.2H7
CD127	Fluidigm	3176004B	A019D5
CD38	Fluidigm	3167001B	HIT2
CXCR3	Fluidigm	3156004B	G025H7
CD45RO	Fluidigm	3165011B	UCHL1
CD95	Fluidigm	3164008B	DX2
CD45RA	Fluidigm	3153001B	HI100
