# Supplementary material for: CD28 costimulation drives tumor-infiltrating T cell glycolysis to promote inflammation
Source: JCI Insight. 2020 Aug 20;5(16):e138729. doi: 10.1172/jci.insight.138729 (PMC7455120; doi:10.1172/jci.insight.138729)
Supplement: Supplemental Table 1 [file jciinsight-5-138729-s088.pdf]

Supplemental Table 1. Patient characteristics

Lab ID	Disease	Subtype	Grade	Age	Sex	Race
197	Renal Cell Carcinoma	Clear Cell	2	58	female	white
198	Renal Cell Carcinoma	Clear Cell	2	60	female	white
213	Renal Cell Carcinoma	Clear Cell	4	59	male	white
215	Renal Cell Carcinoma	Clear Cell	3	55	male	white
220	Renal Cell Carcinoma	Clear Cell	3	44	male	white
227	Renal Cell Carcinoma	Clear Cell	3	62	male	white
228	Renal Cell Carcinoma	Clear Cell	2	67	male	hispanic
240	Renal Cell Carcinoma	Clear Cell	2	62	male	black
248	Renal Cell Carcinoma	Clear Cell	2	66	female	white
251	Renal Cell Carcinoma	Clear Cell	3	67	male	white
252	Renal Cell Carcinoma	Clear Cell	2	44	female	white
254	Renal Cell Carcinoma	Clear Cell	3	48	male	white
257	Renal Cell Carcinoma	Clear Cell	2	43	male	white
263	Renal Cell Carcinoma	Clear Cell	2	63	male	white
269	Renal Cell Carcinoma	Clear Cell	3	67	male	white
271	Renal Cell Carcinoma	Clear Cell	3	65	male	black
274	Renal Cell Carcinoma	Clear Cell	2	68	male	white
277	Renal Cell Carcinoma	Clear Cell	2	47	male	white
283	Renal Cell Carcinoma	Clear Cell	2	55	male	white
287	Renal Cell Carcinoma	Clear Cell	2	74	female	white
292	Renal Cell Carcinoma	Clear Cell	2	71	female	white
293	Renal Cell Carcinoma	Clear Cell	3	34	female	white
295	Renal Cell Carcinoma	Clear Cell	1	65	female	white
298	Renal Cell Carcinoma	Clear Cell	3	68	male	white
301	Renal Cell Carcinoma	Clear Cell	2	66	female	white
305	Renal Cell Carcinoma	Clear Cell	2	44	female	white
307	Renal Cell Carcinoma	Clear Cell	3	52	male	white
309	Renal Cell Carcinoma	Clear Cell	4	76	female	white
314	Renal Cell Carcinoma	Clear Cell	2	63	female	white
315	Renal Cell Carcinoma	Clear Cell	2	68	male	white
317	Renal Cell Carcinoma	Clear Cell	2	57	male	white
322	Renal Cell Carcinoma	Clear Cell	3	72	male	white
323	Renal Cell Carcinoma	Clear Cell	4	84	male	white
324	Renal Cell Carcinoma	Clear Cell	3	48	male	white
325	Renal Cell Carcinoma	Clear Cell	3	56	female	white
326	Renal Cell Carcinoma	Clear Cell	2	64	male	white
328	Renal Cell Carcinoma	Clear Cell	1	62	male	white
333	Renal Cell Carcinoma	Clear Cell	3	43	male	white
334	Renal Cell Carcinoma	Clear Cell	2	65	male	white
337	Renal Cell Carcinoma	Clear Cell	3	58	male	white
339	Renal Cell Carcinoma	Clear Cell	3	66	male	white
341	Renal Cell Carcinoma	Clear Cell	3	40	male	white
342	Renal Cell Carcinoma	Clear Cell	3	51	female	white
345	Renal Cell Carcinoma	Clear Cell	2	45	male	white
