# Supplementary material for: CD28 costimulation drives tumor-infiltrating T cell glycolysis to promote inflammation
Source: JCI Insight. 2020 Aug 20;5(16):e138729. doi: 10.1172/jci.insight.138729 (PMC7455120; doi:10.1172/jci.insight.138729)
Supplement: Supplemental Table 2 [file jciinsight-5-138729-s089.pdf]

Supplemental Table 2. Antibodies for fluorescent flow cytometry

Antigen	Fluorochrome	Clone	Company	Catalog #	Dilution
CD8	eFluor 450	SK1	Invitrogen	48-0087-42	1:400
CD8	PE	RPA-T8	Invitrogen	12-0088-42	1:400
CD25	PerCP-Cy5.5	BC96	Invitrogen	45-0259-42	1:400
CD71	PE	OKT9	Invitrogen	12-0719-42	1:400
CD71	APC	OKT9	Invitrogen	17-0719-42	1:400
Granzyme B	FITC	REA226	Miltenyi	130-118-341	1:200
Glut1	PE	202915	R&D Systems	FAB1418P	1:100
Glut3	FITC	polyclonal	abcam	136180	1:50
IFNy	PECy7	4S.B3	Invitrogen	25-7319-82	1:100
TNFa	eFluor 450	MAb11	Invitrogen	48-7349-42	1:100
IL-2	APC	MQ1-17H12	Invitrogen	17-7029-82	1:100
CD28	APC	CD28.2	Invitrogen	17-0289-42	1:50
