# Supplementary material for: CD28 costimulation drives tumor-infiltrating T cell glycolysis to promote inflammation
Source: JCI Insight. 2020 Aug 20;5(16):e138729. doi: 10.1172/jci.insight.138729 (PMC7455120; doi:10.1172/jci.insight.138729)
Supplement: Supplemental Table 3 [file jciinsight-5-138729-s090.pdf]

Supplemental Table 3. Mass cytometry antibodies.

Target	Provider	Product #	Clone
Glut1	Novus	NB110-39113	Poly
HK II	Abcam	ab131196	1E8-H3-F11
Grim19	Abcam	ab110240	6E1BH7
ATP5a	Abcam	ab110273	7H10BD4F9
CPT1a	Abcam	ab128568	8F6AE9
CytoC	BD Pharmingen	556432	6H2.B4
Glud1	Abcam	ab34786	Poly
CD45	Fluidigm	3141009B	HI30
CD19	Fluidigm	3142001B	HIB19
CD5	Fluidigm	3143007B	UCHT2
CD4	Fluidigm	3145001B	RPA-T4
CD8a	Fluidigm	3146001B	RPA-T8
CD134/OX40	Fluidigm	3150023B	ACT35
CD62L	Fluidigm	3153004B	DREG-56
CD3	Fluidigm	3154003B	UCHT1
CD27	Fluidigm	3155001B	L128
CD69	Fluidigm	3162001B	FN50
CD44	Fluidigm	3166001B	BJ18
CD25	Fluidigm	3169003B	2A3
HLA-DR	Fluidigm	3170013B	L243
Ki-67	Fluidigm	3172024B	B56
Granzyme B	Fluidigm	3173006B	GB11
CD279/PD-1	Fluidigm	3174020B	EH12.2H7
CD127	Fluidigm	3176004B	A019D5
CD38	Fluidigm	3167001B	HIT2
CXCR3	Fluidigm	3156004B	G025H7
CD45RO	Fluidigm	3165011B	UCHL1
CD95	Fluidigm	3164008B	DX2
CD45RA	Fluidigm	3153001B	HI100
